# Chromium (VI)-Induced Alterations in Physio-Chemical Parameters, Yield, and Yield Characteristics in Two Cultivars of Mungbean (*Vigna radiata L*.)

**DOI:** 10.3389/fpls.2021.735129

**Published:** 2021-09-29

**Authors:** Deepti Singh, Nathi Lal Sharma, Chandan Kumar Singh, Vimala Yerramilli, Rup Narayan, Susheel Kumar Sarkar, Ishwar Singh

**Affiliations:** ^1^Department of Botany, Meerut College, Meerut, India; ^2^Division of Genetics, ICAR-Indian Agricultural Research Institute, New Delhi, India; ^3^Department of Botany, Chaudhary Charan Singh University, Meerut, India; ^4^Division of Design of Experiments, ICAR-Indian Agricultural Statistics Research Institute, New Delhi, India

**Keywords:** antioxidant enzymes, chromium, oxidative stress, mungbean, seedling growth, seed yield

## Abstract

Chromium (Cr) presently used in various major industries and its residues possess a potent environmental threat. Contamination of soil and water resources due to Cr ions and its toxicity has adversely affected plant growth and crop productivity. Here, deleterious effects of different levels of Cr (VI) treatments i.e., 0, 30, 60, 90, and 120 μM on two mungbean cultivars, Pusa Vishal (PV) and Pusa Ratna (PR), in hydroponic and pot conditions were evaluated. Germination, seedling growth, biomass production, antioxidant enzyme, electrolytic leakage, oxidative stress (hydrogen peroxide and malondialdehyde), and proline content were determined to evaluate the performance of both cultivars under hydroponic conditions for 15 days. The hydroponic results were further compared with the growth and seed yield attributes of both the genotypes in pot experiments performed over 2 years. Seedling growth, biomass production, total chlorophyll (Chl), Chl-*a*, Chl-*b*, nitrogen content, plant height, seed protein, and seed yield decreased significantly under the 120 μM Cr stress level. Activities of antioxidant enzymes superoxide dismutase, catalase, ascorbate peroxidase and peroxidase increased in the leaves following Cr exposure at 60–90 μM but declined at 120 μM. Cr-induced reductions in growth and seed yield attributes were more in the sensitive than in the tolerant cultivar. Cr accumulation in the roots, stems, leaves, and seeds increased with an increase in Cr concentrations in the pot conditions. Furthermore, for both cultivars, there were significant negative correlations in morpho-physiological characteristics under high Cr concentrations. Overall results suggest that (PR) is more sensitive to Cr stress (PV) at the seedling stage and in pot conditions. Furthermore, (PV) can be utilized to study the mechanisms of Cr tolerance and in breeding programs to develop Cr-resistant varieties.

## Introduction

Industrial and anthropogenic activities increase heavy metal pollution in the environment and adversely affect plant growth and metabolic activity, in addition to yield (Farid et al., 2015; Rizwan et al., 2017; Wakeel et al., 2019). Accumulation of heavy metals (HMs) in edible parts of plants also poses major “risks to humans and animals' health” (Anjum et al., 2016; Shen et al., 2017; Wang et al., 2017). Among HMs, chromium (Cr) is considered the most potent pollutant in ecosystem (Gill et al., 2016a). Chromium, which has a specific density of 7.19 g/cm^3^, ranks seventh in the list of the most abundant metals and is the 21st most abundant HM in the Earth's crust (Economou-Eliopoulos et al., 2013). The most common oxidation states of Cr are Cr (III) and Cr (VI) (Ashraf et al., 2017; Choppala et al., 2018). Of which, Cr (VI) is more mobile and toxic to living organisms, with mutagenic and carcinogenic effects (Beyersmann and Hartwig, 2008). The higher toxicity of Cr (VI) can be attributed to its high solubility, which increases the incidence of gastric and lung cancers significantly (Zhu et al., 2019). High Cr contents in the soil get absorbed and accumulated in the above-ground parts of plants and subsequently enters the food chain, in turn directly or indirectly affecting human health (Giri and Singh, 2017). Further, it negatively effects the plants by seizing it's growth, enzyme activity, photosynthesis, metabolism, biomass production, and crop productivity (Anjum et al., 2017; Singh et al., 2020). Uptake of Cr (VI) by roots are through phosphate and sulfate pathways (Gill et al., 2017). Thereafter, it is easily transported to other parts of the plant, whereas Cr (III) is preferably transported through an inactive pathway (Cornelis et al., 2005; Park, 2020). Since roots are the first plant organs to be exposed to Cr contamination in the soil, they play a primary role in its accumulation and translocation (Jaison and Muthukumar, 2017; Zhao et al., 2019; Zong et al., 2020).

Phyto-toxic effects of excess Cr result in reduced growth, photosynthesis, mineral nutrients, and crop productivity (Sehrish et al., 2019; Singh et al., 2020). Cr toxicity also affects other physiological processes, such as seed germination and enzyme activity (López-Bucio et al., 2014; Singh et al., 2020; Wakeel and Xu, 2020). Dotaniya et al. (2014) reported that elevated Cr accumulation in plants reduced germination and root and shoot growth rate, in turn affecting total biomass, and ultimately, plant yield. Cr toxicity in plant systems and its physiological modulation mainly depend on the quantity of Cr uptake by a plant, its mobilization, and subsequent accumulation in various tissues (Hayat et al., 2012). In addition, the uptake of Cr from the rhizosphere by plants is usually accompanied by water and nutrients uptake, resulting in many variations in morpho-physiological, biochemical processes and molecular levels (Wakeel et al., 2018, 2019; Farid et al., 2019). Shunted nutrient uptake/translocation may be due to weaker Cr competitive binding capacity with carrier channels and plasma membrane H^+^ ATPase activity (Shahid et al., 2017). A recent study described the impact of Cr phytotoxicity on morpho-physiological attributes such as root and shoot length, root and shoot biomass, chlorophyll contents, metabolic antioxidants, stress markers (malondialdehyde, hydrogen peroxide), relative water content, proline, electrolyte leakage (EL) and total Cr uptake in *Sesbania Sesban* (Din et al., 2020), *Cicer arietinum* (Singh et al., 2020), and *Spinacia oleracea* (Zaheer et al., 2020).

Furthermore, Cr accumulation at the cellular level triggers the production of reactive oxygen species (ROS), resulting in oxidative damage, as indicated by malondialdehyde (MDA), hydrogen peroxide (H_2_O_2_) and electrolyte leakage (EL) formation (Yu et al., 2018; Patra et al., 2019). The oxidative damage caused due to Cr (VI) toxicity is more pronounced than that associated with Cr (III) toxicity (Beyersmann and Hartwig, 2008). Stress alters cellular ROS homeostasis leading to ROS accumulation (Singh et al., 2013; Maqbool et al., 2018; Zaheer et al., 2019). ROS accumulation causes peroxidation of membrane lipids, which leads to the disruption of membrane structure and function, along with the oxidation of proteins and nucleic acids (Rai et al., 2014; Zhang et al., 2016; Wakeel et al., 2019). ROS accumulation also inhibits the plant biochemical responses, resulting in altered morphology and architecture (Farid et al., 2017; Li et al., 2018; Wakeel et al., 2020). To cope with the deleterious effects of ROS production, plants have scavenging mechanisms comprising of antioxidant enzymes viz. peroxidase (POD), catalase (CAT), superoxide dismutase (SOD), and ascorbate peroxidase (APX) (Zaheer et al., 2020). However, Cr toxicity also affects the activities of the enzymes (Gill et al., 2015b; Wakeel et al., 2020). Overall, Cr accumulation at high concentrations would alter the biochemical and physiological attributes of plants and ultimately lead to low productivity and yield (Anjum et al., 2017; Singh et al., 2020). Consequently, plants have had to evolve appropriate defense mechanisms to counter Cr toxicity and oxidative losses induced by Cr accumulation.

Industrial and tannery waste from the leather and other industries are a major source of Cr pollution in many parts of India, such as in Ghaziabad, Ranipet, Kanpur, Vadodara, and Talcher, as well as globally (Suthar et al., 2009; Zaidi and Pal, 2017). The Hindon River water and sediment in the Ghaziabad region, Uttar Pradesh, are the most polluted, and have the highest Cr concentrations (Suthar et al., 2009; Singh and Sharma, 2018). In the Ghaziabad region, various industries use Cr compounds in their manufacturing activities. The declining quality of the Hindon River water and its surrounding soil is a major source of concern, particularly with regards to the risks posed to human life, as the rural population of Western Uttar Pradesh, India, is dependent on the water source for various activities. It also threatens other flora and fauna in the region, as well as ecological and food production systems. The screening and breeding of crop cultivars with relatively low Cr accumulation potential are some of the strategies that could be adopted to facilitate sustainable food production in the wake of increased Cr pollution in the environment in such regions.

Mungbean (*Vigna radiata* L.) is an important legume crop that grows optimally under drought conditions but is negatively affected by Cr toxicity (Jabeen et al., 2016). Two mungbean cultivars were selected for the present study: Pusa Vishal (PV), which is a tolerant cultivar, and Pusa Ratna (PR), which is a sensitive cultivar for drought tolerance (Jisha and Puthur, 2014). Although the effects of NaCl and poly-ethylene glycol (PEG) toxicity on both mungbean cultivars have been reported, no studies have been conducted on the sensitivity of the two cultivars to Cr toxicity. Screening of Cr tolerance in plants has been done under hydroponic, pot and field conditions (Xu et al., 2018). Of which, hydroponics is much efficient and reliable technique as (i) it can screen plants at very early growth phases; (ii) it enables screening of large number of genotypes with stringent control over pH and nutrient availability to plants; (iii) it is a non-destructive screening strategy (v) further, after initial evaluation for Cr tolerance, plants can be shifted back to fields to study successive growth stages. Whereas, under pot and field conditions, reliable and consistent results are hard to find, due to unevenness of Cr content. However, plants response toward Cr stress under hydroponic conditions was also found to be well-correlated under soil-based screening (Macarisin et al., 2014; Park, 2020). The objectives of the present study were to (i) investigate the effect of Cr (VI) on the germination and seedling growth of the two mungbean cultivars based on MDA, H_2_O_2_, and proline contents, EL, and enzyme activities under hydroponic conditions; (ii) compare the morpho-physiological and yield attribute responses of both cultivars to Cr stress and examine Cr accumulation in various parts (roots, stems, leaves, and seeds) of the mungbean cultivars under pot conditions.

## Materials and Methods

### Plant Material

The seeds of both cultivars i.e., Pusa Vishal (PV) and Pusa Ratna (PR) were obtained from the Division of Seed Technology, Indian Council of Agricultural Research-Indian Agriculture Research Institute, Pusa, New Delhi, India.

### Evaluation of Cultivars Under Different Experimental Conditions

#### Germination Experiments

Healthy and similar sized seeds were sorted and sterilized with 0.2% (w/v) mercuric chloride (HgCl_2_) for 5 min. Seeds were then washed with distilled water and soaked for 4 h. Mungbean seeds were placed on germination paper in petri dishes to measure germination. Five hexavalent Cr concentrations i.e., 0, 30, 60, 90, and 120 μM were applied to assess the germination and used distilled water as a control. Twenty seeds were placed in each Petri dish and then incubated in a biological oxygen demand chamber (IEC−60601, Waiometra, Delhi, India) set at 25 ± 2°C and a relative humidity of 75–80%. Seed germination percentage was recorded after 48 h. Percentage germination was calculated using the formula of Akinci and Akinci (2010). Each treatment had three replicates.

#### Hydroponic Experiments

After 5 days, healthy seedlings of similar sizes were selected and planted in a plastic container (10 L) with nutrient solution. The nutrient composition was prepared according to Simon et al. (1994). Each seedling was then exposed to Cr stress at five varying concentrations of Cr (VI) as mentioned in previous section. The hydroponic test was conducted under controlled environmental conditions in the growth chamber at the National Phytotron Facility (NPF), New Delhi, India. The control conditions were maintained with air temperature of 24/20°C (day/night; ± 2°C), a photoperiod of 10/14 h Day/Night, and relative humidity 60%. The experiment duration was 15 days. The experimental design was a completely randomized design (CRD) with three relications. Growth parameters like root and shoot length and fresh as well as dry weight of root and shoot were also measured. Further, physiological and biochemical attributes were also determined under control and Cr stress.

#### Morphological Parameters

Root and shoot length of both the cultivars were measured manually under control and Cr stress conditons. Subsequently, root and shoot fresh weight were measured. Therafter, root and shoot were kept in oven at 80°C to measure the dry weight.

### Physio-Biochemical Parameters

#### Assessment of Malondialdehyde (MDA) Contents

The level of MDA was estimated in control and Cr exposed root and shoot of both the cultivars following the protocol described by Heath and Packer (1968). Root and shoot samples (0.25 g each) were homogenized in 5 ml of 0.1% trichloro acetic acid (TCA). The homogenate was centrifuged at 10,000 × g for 10 min thereafter, 1 ml aliquot of the supernatant was added with 4 ml of 20% TCA containing 0.5% thiobarbituric acid (TBA). The mixture was then heated at 95°C for 30 min, and immediately cooled in an ice bath. It was followed with a centrifugation at 10,000 × g for 30 min, and absorbance of the supernatant at 532 and 600 nm. The MDA contents were calculated by applying an extinction coefficient of 155 mM^−1^ cm^−1^.

#### Assessment of Hydrogen Peroxide (H_2_O_2_) and Electrolyte Leakage (EL) Contents

The hydrogen peroxide (H_2_O_2_) contents were determined according to Jana and Choudhuri (1981). Hydrogen peroxide was extracted by homogenizing 50 mg of root and shoot tissue samples in 10 ml of phosphate buffer (50 mM, pH 7.1), that contained a catalase inhibitor, hydroxylamine (1 mM). The homogenate was centrifuged at 6,000 × g for 15 min. A mixture comprised of 3 ml of extracted solution and 1 ml of 0.1% titanium sulfate in 20% (v/v) H_2_SO_4_ was centrifuged at 6,000 × g for 10 min. The intensity of the yellow color of the supernatant was measured at 410 nm. The H_2_O_2_ level was calculated, using the extinction coefficient 0.28 μmol^−1^ cm^−1^.

The electrolyte leakage was measured in roots and shoots of control and Cr exposed plants using procedures mentioned in Valentovic et al. (2006). The samples were washed and place in tubes filled with 10 ml deionized water. These tubes were then incubated in a water bath at 30°C for 2 h to denote initial electrical conductivity (EL_1_). Further, tubes were again incubated at 90°C for 90 min to measure the final electrical conductivity (EL_2_) and electrical conductivity (EL%) was estimated using by the following equation.


$$\begin{array}{l} \text{EL}=\text{E}{\text{L}}_{1}/\text{E}{\text{L}}_{2}\times 100 \end{array}$$

### Proline Contents

Proline contents were determined according the methodology of Bates et al. (1973). Sulfosalicylic acid (3%, 5 ml), was used to homogenize 0.5 g of root and shoot tissues, followed by centrifugation at 5,000 × g for 10 min. Supernatant (2 μL) was decanted and mixed with 2 ml acid-ninhydrin and 2 ml acetic acid. Reaction mixtures were boiled at 90°C for 60 min followed by cooling on ice and final extraction with toluene (5 ml). Proline content was determined based on three replicates as μmol·g^−1^ fresh weight (FW), by obtaining absorbance values at 520 nm.

### Antioxidant Enzyme Activity

After 15 days of treatment, the top fresh leaves of both cultivars were used to determine antioxidative enzyme activity. Fresh leaves were (1.0 g) taken and ground with 0.5 M phosphate buffer (pH 7.20), in a pre-cooled pestle and mortar. The homogenized sample was filtered using muslin cloth and centrifuged at 12,000 × g at 4°C for 10 min. The supernatant was collected and used for the estimation of SOD and POD antioxidant enzyme activities. Antioxidant enzyme activities were determined according to Zhang (1992).

Catalase (CAT) enzyme activity was determined according to Aebi (1984). The assay mixture (3 ml) was prepared using enzyme extract (100 μL), 2.8 ml potassium phosphate buffer (50 mM, pH-7.0), and 100 μl H_2_O_2_ (300 mM), with 2 mM ethylenediaminetetraacetic acid disodium salt (EDTA-Na_2_). The CAT enzyme activity was evaluated from the decline in absorbance at 240 nm due to the loss of H_2_O_2_.

Ascorbate peroxidase (APX) enzyme activity was determined according to Nakano and Asada (1981). APX enzyme activity was estimated from a (3 ml) mixture solution containing 100 μl enzyme extract, 100 μl H_2_O_2_ (300 mM), 2.7 ml potassium phosphate buffer (25 mM), and 100 μl ascorbate (7.5 mM) with 2 mM EDTA (pH-7.0). APX enzyme activity was measured by monitoring the variations in the wavelength at 290 nm.

### Pot Experiment

Pot experiments were carried out for both cultivars in the botanical garden of Meerut College, Meerut, Uttar Pradesh, India, in 2017–2018, and 2018–2019. The pot trials were arranged in a CRD with seven pots per treatment. Cr treatments of 0, 30, 60, 90, and 120 μM was then applied in the form of K_2_Cr_2_O_7_ to each pot. Sandy loam soil (5 kg) was filled into each PVC pot. The basic organic properties of the sandy loam are listed in Supplementary Table S1, as reported by Singh et al. (2020). Mungbean seeds were sown by hand in each pot and were thinned to four uniform plants per pot, 5 days after germination. After 12 days of germination, N, phosphorous, and potassium fertilizer was applied at rates of 2.30, 0.5, and 2.15 g·kg^−1^ in each pot, respectively. After 15 days of sowing, each pot was irrigated with different Cr solutions at 1-week intervals.

### Chlorophyll and Nitrogen Contents

Thirty-five days after sowing (DAS), chlorophyll content (Chl-*a*, Chl-*b*, and total Chl) was measured in the fresh uppermost leaves of both plants. The leaf chlorophyll supernatant was extracted using acetone (85% v/v) and kept in the dark at 4°C until the leaves decolorised, and then centrifuged at 3,000 × g for 10 min at 4°C. Absorbance values of the supernatants were obtained at 663 nm and 644 nm for chlorophyll-a and b, respectively, estimated using a UV-1800 spectrophotometer (Shimadzu, Japan).

Total chlorophyll content was detemined using the following formula:


$$\begin{array}{l} \text{Total chlorophyll}(\text{μg}\cdot {\text{g}}^{-\text{1}}\text{ fresh leaf})\text{ = }[\{(\text{20}.\text{2}\times A_{\text{644}})\\ \text{+}(\text{8}.\text{02}\times A_{\text{663}})\}/\text{1000}\times W]\times V, \end{array}$$

where, *W* = FW and *V* = extraction volume.

Chlorophyll contents was measured according to Arnon (1949). Nitrogen (N) content in the root and shoot tissues were measured 35 DAS using the micro-Kjeldahl method (Iswaran and Marwah, 1980). N content was measured using the previous technique described by Singh et al. (2020).

### Plant Growth and Yield-Related Parameters

Nine-week-old plants were harvested and thoroughly washed using tap water and double-distilled water. Growth parameters, such as number of primary and secondary branches per plant, plant height, and biomass were determined. To measure dry biomass, fresh biomass of each plant was dried at 70°C for 12 h. For yield attributes, pods per plant, seeds per pod and as well as seed yield per plant, and hundred seed weight were measured.

#### Grain Protein Content

After 9 weeks of sowing, plants were harvested and total protein contents in seeds were estimated according to Lowry et al. (1951) method. Seeds (500 mg) of each cultivar were ground in 5 ml phosphate buffer (pH 7.20) after soaking in the buffer. Total grain protein was extracted using 0.1 M NaOH and the extract was reacted with Folin phenol reagent to develop a blue colored compound. Total grain protein content (mg·g^−1^ FW) was estimated using a Bovise Serum Albumin standard curve.

### Estimation of Chromium Content

Chromium content was quantified 63 DAS. Root, leaf, stem, and seed samples (0.5 g each) were heated in 15 ml conc. HNO_3_:HClO_4_ (3:1, v/v) on a hot plate, by gradually increasing the temperature up to 275°C till yellow fumes began to emerge from the flask. H_2_O_2_ was added once the density of fumes decreased. After cooling, distilled water was added to the samples to achieve a total volume of 25 ml. Cr content was measured by obtaining absorbance values using a ZEEnit 700 P atomic absorption spectrometer (Analytik Jena AG, Germany).

#### Statistical Analyses

After determining the impact of varying Cr treatment levels on physio-chemical characteristics, and yield components of both Mungbean cultivars, the results were described as mean value ± standard error. The replicated data were statistical analysis using one-way analysis of variance (ANOVA) using SAS® version 9.4, and means were compared by the LSD_0.05_ test. Correlations among different attributes at different phases of growth were explored by calculating Pearson's correlation coefficient. Graphics were created using R software and Statgraphics 18. For all measures, only *p* < 0.05 were considered significant.

## Results

### Seed Germination

**Figure 1:**
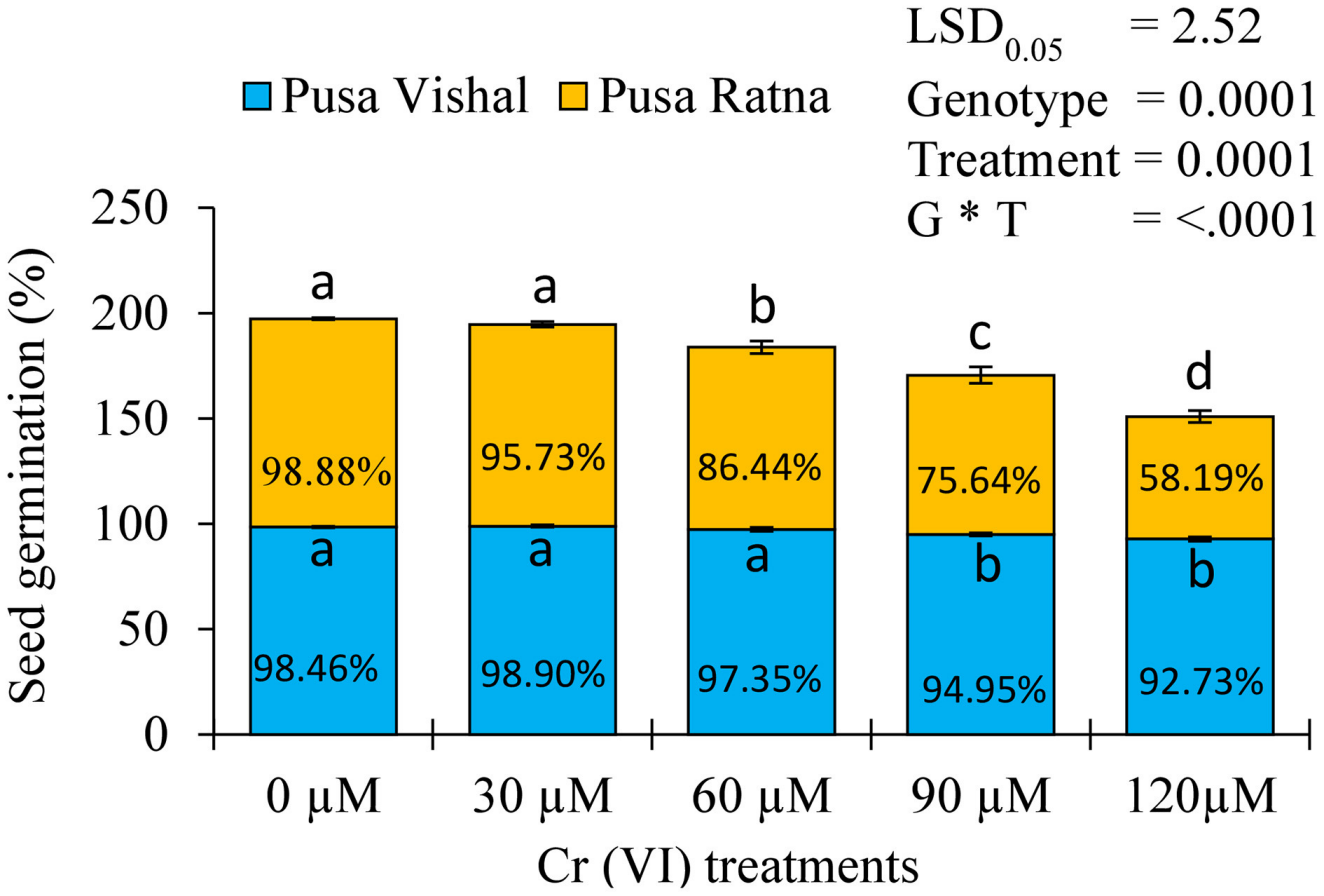
Percent inhibition of germination in the two Mungbean cultivars under chromium (Cr [VI]) toxicity. Mean values of three replicates are shown; error bar (± standard error [SE]). Mean values with the same letter don't have significant difference at *p* ≤ 0.05, according to the Least Significant Difference test (LSD_0.05_).

**Figure 2:**
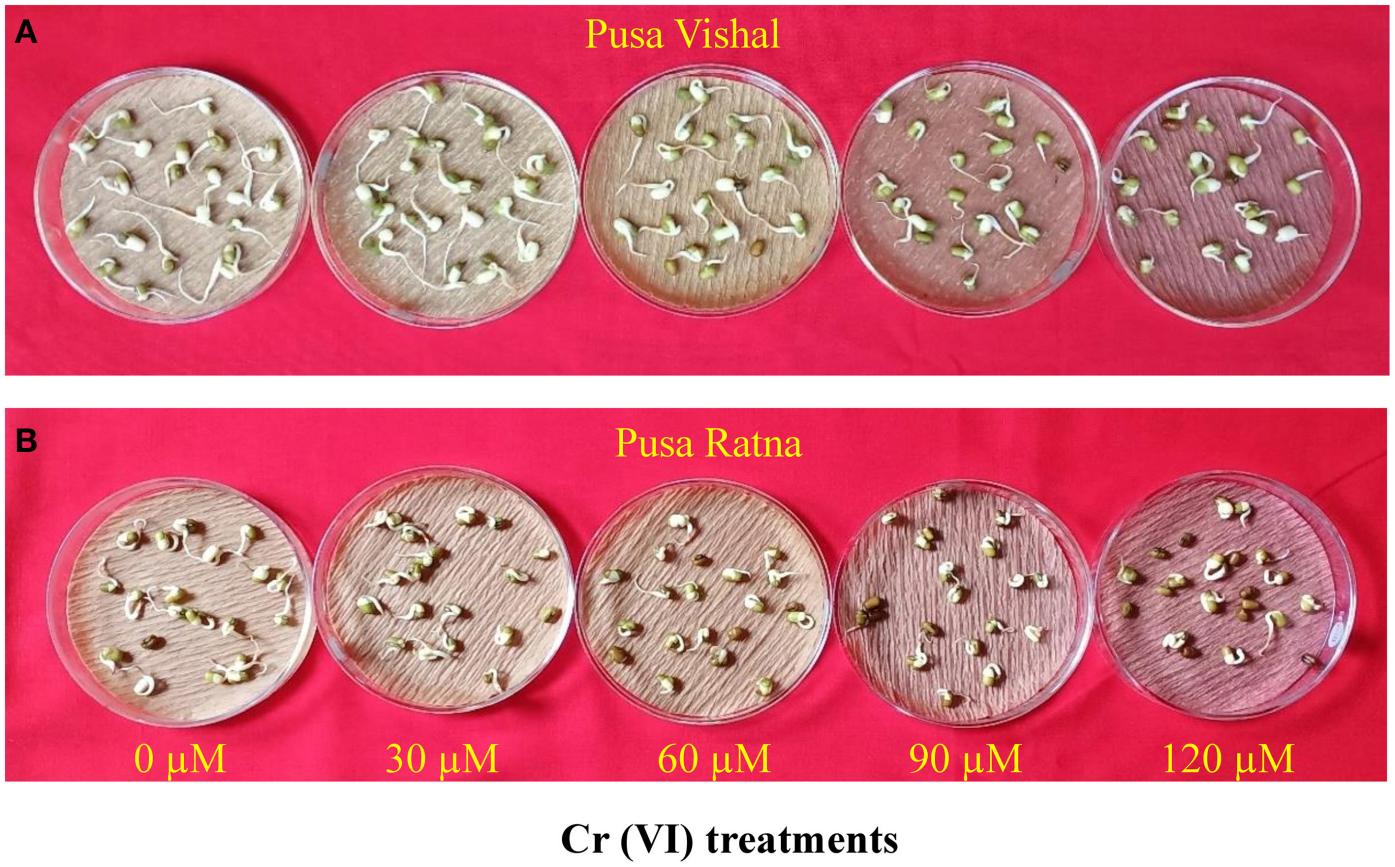
Effect of different chromium (Cr [VI]) concentrations on seed germination in two Mungbean cultivars, **(A)** Pusa Vishal (PV) and **(B)** Pusa Ratna (PR).

Cr (VI) significantly affected the germination of the two cultivars. The reductions in germination were greater in PR (45%) than in PV (41%) under all the Cr (VI) toxicity levels examined (Figure 1). PR exhibited the maximum germination injury under the 120 μM Cr treatment, whereas Pusa Vishal exhibited minimal injury under the treatment (Figure 2). Overall, PV exhibited greater tolerance than PR (Figures 1, 2), even though both cultivars had almost similar germination rates in Petri dishes in the control condition.

### Effect of Cr (VI) on Morpho-Physiological Attributes Under Hydroponic Conditions

**Figure 3:**
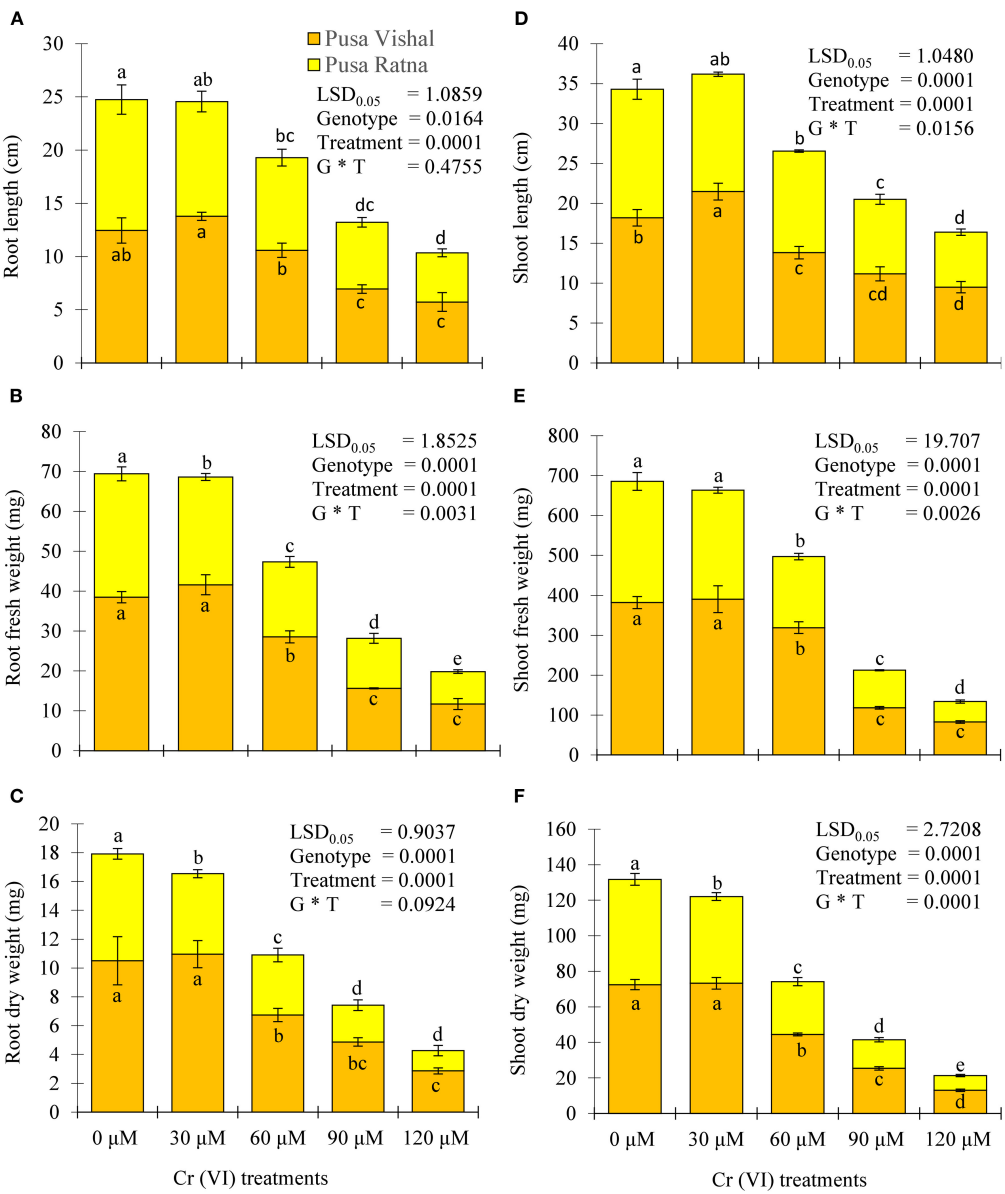
Effect of different chromium (Cr [VI]) concentrations on **(A)** root length, **(B)** root fresh wieght **(C)** root dry weight, **(D)** shoot length, **(E)** shoot fresh wieght, and **(F)** shoot dry weight of two Mungbean cultivars, Pusa Vishal (PV) and Pusa Ratna (PR), grown in hydroponic conditions. Mean values of three replicates are shown; error bar (± standard error [SE]). Mean values with the same letter don't have significant difference at p ≤ 0.05, according to the Least Significant Difference test (LSD_0.05_).

**Figure 4:**
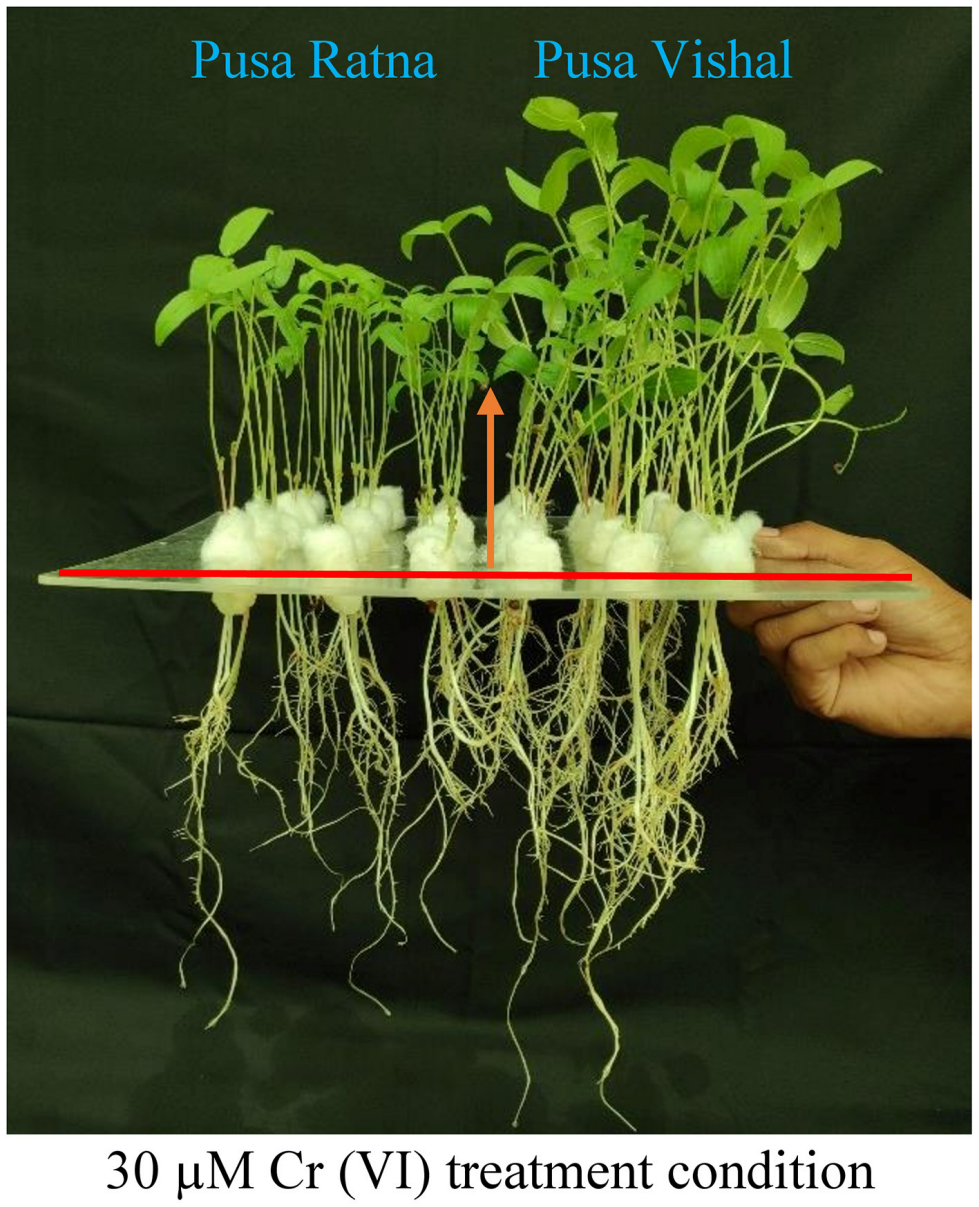
Effect of low chromium (Cr [VI]) treatment level (30 μM) on the growth of two Mungbean cultivar, Pusa Vishal (PV) and Pusa Ratna (PR) seedlings grown under hydroponic conditions.

Visual symptoms of Cr (VI) toxicity were observed in both Mungbean cultivars. After 15 days of treatment, the morpho-physiological attributes were evaluated using four parameters: length of root and shoot, fresh and dry weight of roots and shoot. Morpho-physiological characteristics (root length, shoot length, fresh and dry weight of the root and shoots) of Mungbean plants markedly decreased under the 120 μM Cr stress level (Figure 3), when compared to those in the control. The Cr stress significantly reduced the root and shoot length by 54% and 48% in PV, and 62.44%, 57.23% in PR, respectively. Under Cr-stress having same treatment condition, reduction in the fresh weight of roots and shoot were 70% and 78.35% in PV, while, 74% and 83.15% in PR, respectively. Similarly, dry weight of root and shoot of PV was decreased by 73% and 82.06%, whereas for PR reduction was 81% and 86%, respectively. In addition, PV showed improved seedling growth and biomass under the 30 μM Cr (VI) treatment compared to those in the control (Figure 4), while there were declines in PR under the same treatment (Figure 4). The results clearly indicated that PV morpho-physiological characteristics less declined than PR.

### Oxidative Stress and Proline Content

Increasing Cr levels induced oxidative stress by increasing EL, MDA and H_2_O_2_ content in both Mungbean cultivars. High Cr concentrations in the nutrient solution increased EL, H_2_O_2_ and MDA levels in Mungbean root and shoot significantly, in comparison to in the control. At 120 μM Cr stress level, increase in the MDA content of root and shoot was 60% and 65.13% in PV, and 68% and 72.32% in PR, when compared to control. Under the same treatment condition, the H_2_O_2_ content increased in root and shoot by 36% and 36% for PV, and 49.05% and 48% for PR. Similarly, the EL of root and shoot increased by 55% and 55% in PV while 68% and 71.19% in PR, respectively. These results showed that the EL, MDA content and H_2_O_2_ levels in the roots and shoots significantly increased under Cr stress both cultivars. However, in PV, these parameters were not significantly different from those under the low Cr (30 μM) concentration and when compared to those in the control (Table 1). Moreover, oxidative stress was prominent in PR as compared to PV and were also indicated by elevated H_2_O_2_ and MDA content (Table 1). Data showed that under 120 μM Cr treatment level, the proline content in root and shoot of PV increased by 72% and 63%, respectively, compared to the control. Similarly, the proline content of root and shoot increased by 50.46% and 42% in PR, respectively. Furthermore, proline contents increased in the roots and shoots of both cultivars under Cr stress and were higher in PV as compared to PR (Supplementary Figure 1).

**Table 1:** Effect of different treatment levels of Cr (VI) on malondialdehyde (MDA, μmol·g^−1^ fresh weight) and hydrogen peroxide (H_2_O_2_, μmol·g^−1^ fresh weight) contents and electrolytic leakage (EL %) in the roots and shoots of two Mungbean cultivars, Pusa Vishal (PV) and Pusa Ratna (PR).

**Cultivars**	**Cr levels**	**MDA**		**H_2_O_2_**		**EL%**	
		**Root**	**Shoot**	**Root**	**Shoot**	**Root**	**Shoot**
Pusa Vishal	0 μM	8.44 ± ±0.23^d^	6.22 ± 0.12^d^	74.86 ± 0.20^d^	67.95 ± 0.24^d^	40.94 ± 0.25^d^	32.21 ± 0.20^d^
	30 μM	8.79 ± 0.13^d^	6.32 ± 0.10^d^	75.07 ± 0.18^d^	68.54 ± 0.41^d^	41.16 ± 0.31^d^	30.59 ± 0.29^e^
	60 μM	12.91 ± 0.11^c^	10.00 ± 0.8^c^	87.04 ± ±0.32^c^	75.28 ± 0.20^c^	62.65 ± 0.26^c^	53.11 ± 0.35^c^
	90 μM	16.11 ± 0.12^b^	13.05 ± 0.10^b^	103.15 ± 0.34^b^	92.77 ± 0.19^b^	80.77 ± 0.19^b^	74.07 ± 0.27^b^
	120 μM	20.85 ± 0.22^a^	17.84 ± 0.23^a^	116.15 ± 0.38^a^	106.04 ± 0.14^a^	95.88 ± 0.29^a^	87.09 ± 0.11^a^
	**LSD**_**0.05**_	**0.54**	**0.43**	**0.93**	**0.80**	**0.83**	**0.81**
Pusa Ratna	0 μM	8.36 ± 0.25^e^	5.86 ± 0.13^e^	72.80 ± 0.18^e^	63.93 ± 0.24^e^	39.37 ± 0.26^e^	30.88 ± 0.31^e^
	30 μM	9.78 ± 0.17^d^	7.82 ± 0.19^d^	83.26 ± 0.14^d^	75.11 ± 0.13^d^	55.94 ± 0.25^d^	43.10 ± 0.30^d^
	60 μM	14.90 ± 0.16^c^	11.97 ± 0.15^c^	98.37 ± 0.33^c^	88.12 ± 0.12^c^	86.05 ± 0.47^c^	65.75 ± 0.29^c^
	90 μM	19.91 ± 0.10^b^	16.16 ± 0.14^b^	116.23 ±±0.40^b^	102.93 ± 0.34^b^	105.23 ± 0.26^b^	96.30 ± 0.23^b^
	120 μM	26.04 ± 0.22^a^	21.17 ± 0.16^a^	142.88 ±±0.34^a^	121.99 ± 0.39^a^	121.90 ± 0.26^a^	108.30 ± 0.24^a^
	**LSD**_**0.05**_	**0.59**	**0.50**	**0.92**	**0.84**	**0.98**	**0.87**

*Mean values of three replicates are shown; error bar (± standard error [SE]). Mean values with same letter don't have significant difference at p ≤ 0.05 according to the Least Significant Difference test (LSD_0.05_). The bold value in the table are least significant difference (0.05)*.

### Antioxidant Enzyme Activity

**Figure 5:**
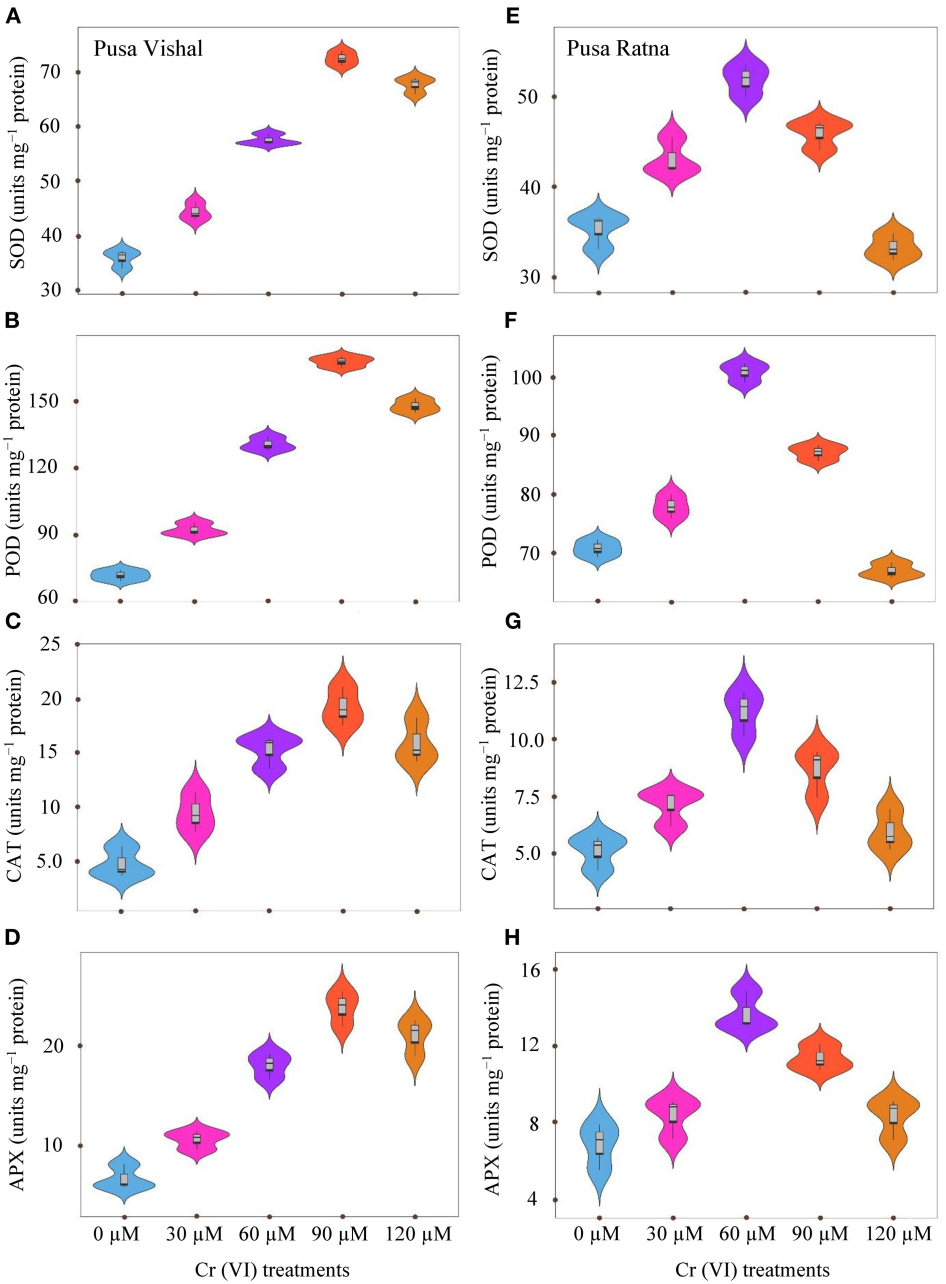
Violin plots screening the distribution of values for superoxide dismutase (SOD) **(A,E)**, ascorbate peroxidase (APX) **(D,H)**, peroxidase (POD) **(B,F)**, and catalase (CAT) **(C,G)**, in leaves of Pusa Vishal (PV) and Pusa Ratna (PR) cultivars grown in hydroponic conditions with different Cr (VI) concentrations.

Activities of CAT, SOD, APX and POD in the leaves of both Mungbean cultivars were higher under Cr stress than in the control (Figure 5). The enzyme activities of SOD, POD, CAT and APX were decreased by 6, 5.37, 14.26, and 18% in PR under 120 μM Cr treatment level, with respect to their controls. Whereas, in PV it increased by 47, 51.44, 70.04, and 68%, respectively. Results also showed that the enzyme activity consistently increased in the leaves of PR up to 60 μM Cr and then declined under the 90–120 μM Cr treatments (Figures 5E–H). Enzyme activity in PV significantly increased upto 90 μM Cr treatment and decreased under the 120 μM Cr stress level (Figures 5A–D). However, enzyme activity in the leaves of PR was lower than in PV under all Cr concentrations. Overall, the results indicate that antioxidant enzyme activity was more affected in the leaves of PR than in the leaves of PV under Cr toxicity.

### Plant Growth and Yield Performance Under Pot Experiments

**Figure 6:**
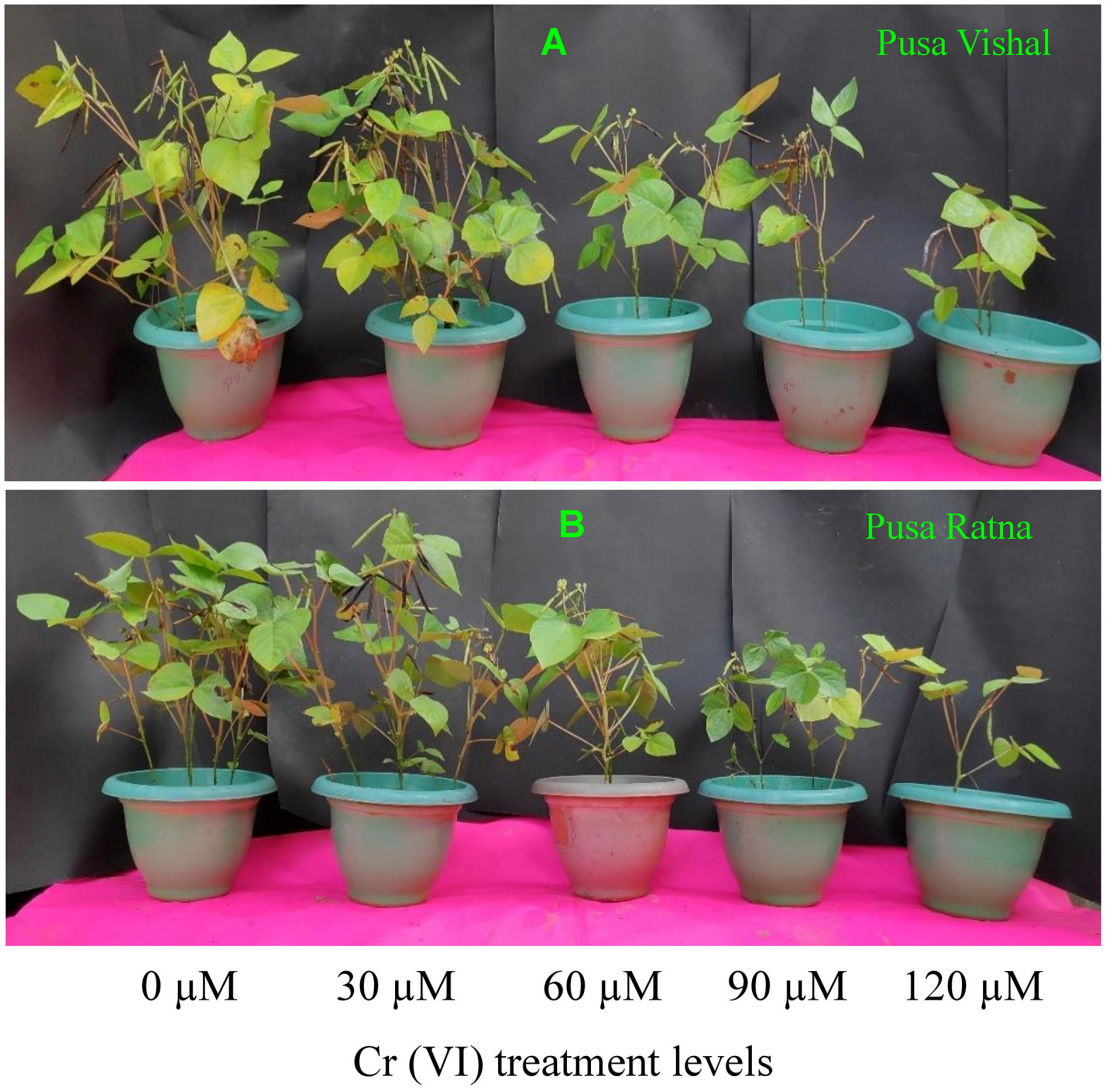
Effect of different chromium (Cr [IV]) treatments on growth, yield and yield-components of two Mungbean cultivars, **(A)** Pusa Vishal (PV) and **(B)** Pusa Ratna (PR).

**Figure 7:**
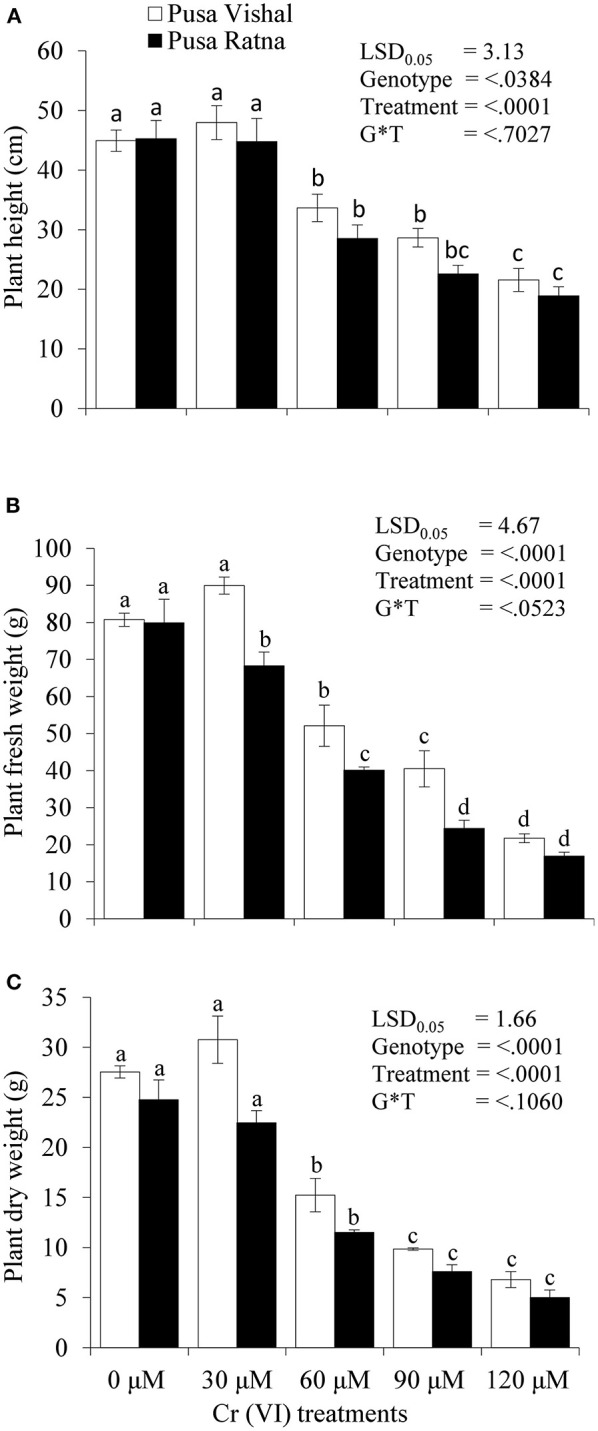
Effect of different chromium (Cr [IV]) treatments on: **(A)** plant height (cm), **(B)** plant fresh weight (g), and **(C)** plant dry weight (g) of two Mungbean cultivars, Pusa Vishal (PV) and Pusa Ratna (PR) in pot conditions. Mean values of three replicates are shown; error bar (± standard error [SE]). Mean values with same letter don't have significant difference at p ≤ 0.05 according to the Least Significant Difference test (LSD_0.05_).

**Figure 8:**
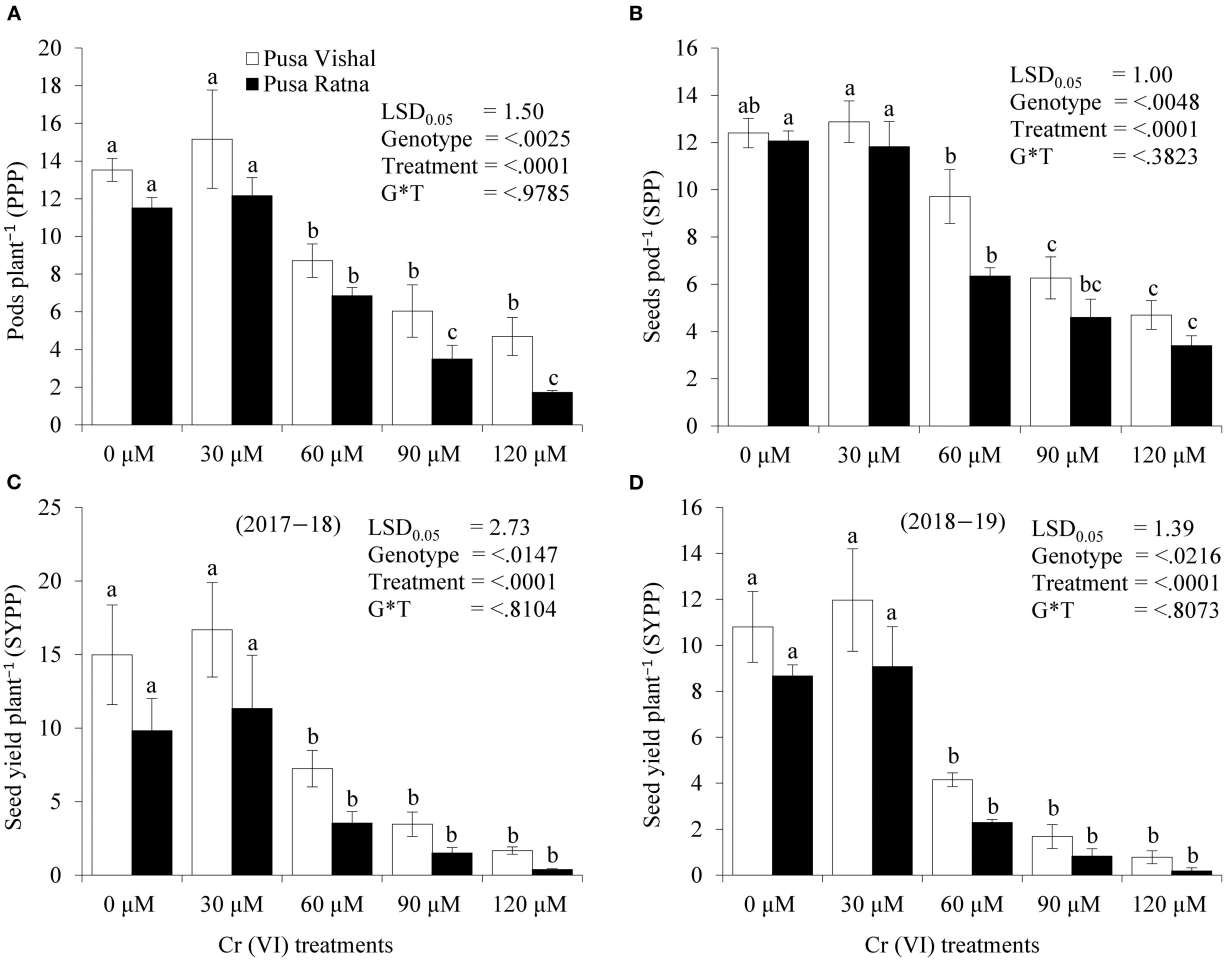
Effect of different chromium (Cr [VI]) treatments on yield attributes:—**(A)** Pods per plant, **(B)** Seeds per pods, **(C)** Seed yield per plant (2017–2018), and **(D)** Seed yield per plant (2018–2019) in two Mungbean cultivars, Pusa Vishal (PV) and Pusa Ratna (PR), grown in pot conditions. Mean values of three replicates are shown; error bar (± standard error [SE]). Mean values with the same letter don't have significant difference at p ≤ 0.05 according to the Least Significant Difference test (LSD_0.05_).

**Figure 9:**
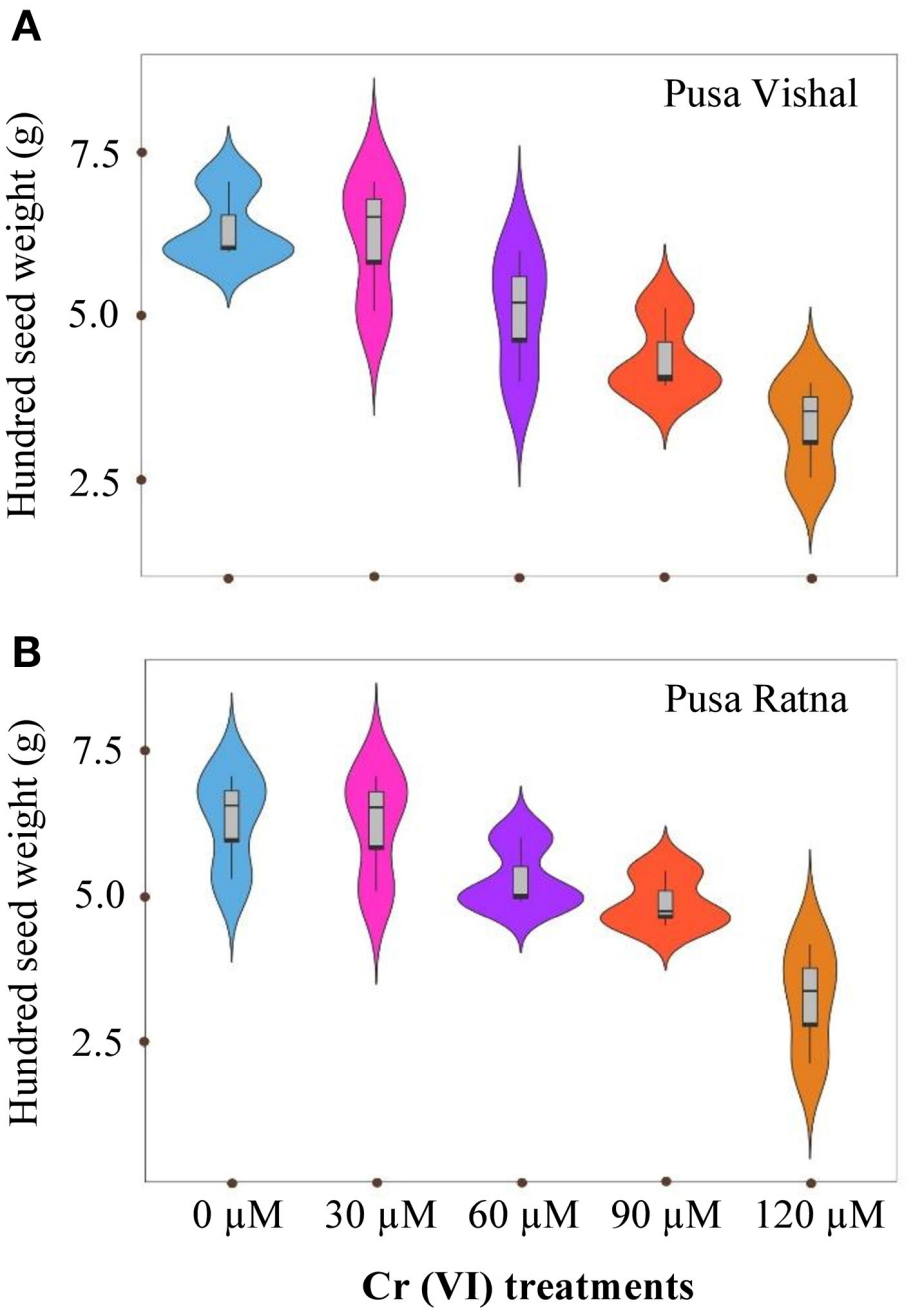
Violin plots screening the distribution of values for hundred seed weight (HSW) in two Mungbean cultivars grown in pot conditions under chromium (Cr [VI]) stress. **(A)** Pusa Vishal (PV) and **(B)** Pusa Ratna (PR).

The effect of Cr stress on growth, yield, and yield attributes of Mungbean plants are illustrated in Figure 6. Both PV and PR exhibited significant reduction in growth attributes (plant height, primary and secondary branches per plant, plant fresh and dry biomass) under the 120 μM Cr level when compared to those in the control (Figure 7 and Supplementary Figure 2). Exposure to Cr stress reduced growth attributes i.e., plant height by 36.22%, primary and secondary branches per plant by 68.03% and 70%, followed by plant fresh and dry biomass 70% and 73.15%, in cultiver PV. Simililarly, PR showed 50.10, 69.17, 75.45, 75, and 73.50% plant height, primary and secondary branches per plant, plant fresh and dry biomass, respectively. Additionally, yield and yield-related attributes such as number of pods per plant, seeds per pod, and 100-seed weight (HSW) were significantly affected in both cultivars under Cr toxicity (Figures 8, 9). Seed yield per plant of PV and PR decreased by 89% and 96.13% under the 120 μM Cr level in 2017–2018 (Figure 8C). Similar results were also observed in 2018–2019 (Figure 8D). During two consecutive years, grain yield was higher at low Cr concentrations in both cultivars than in the control (Figures 8C,D). Moreover, results showed that the Plant growth and development decreased with an increase in Cr concentrations, whereas parameters related to growth, yield, and yield attributes were enhanced under low Cr level (30 μM) in both cultivars. Thus, the overall performance of PV was superior to that of PR with regards to growth, yield, and yield attributes.

### Chlorophyll and Nitrogen Contents

**Figure 10:**
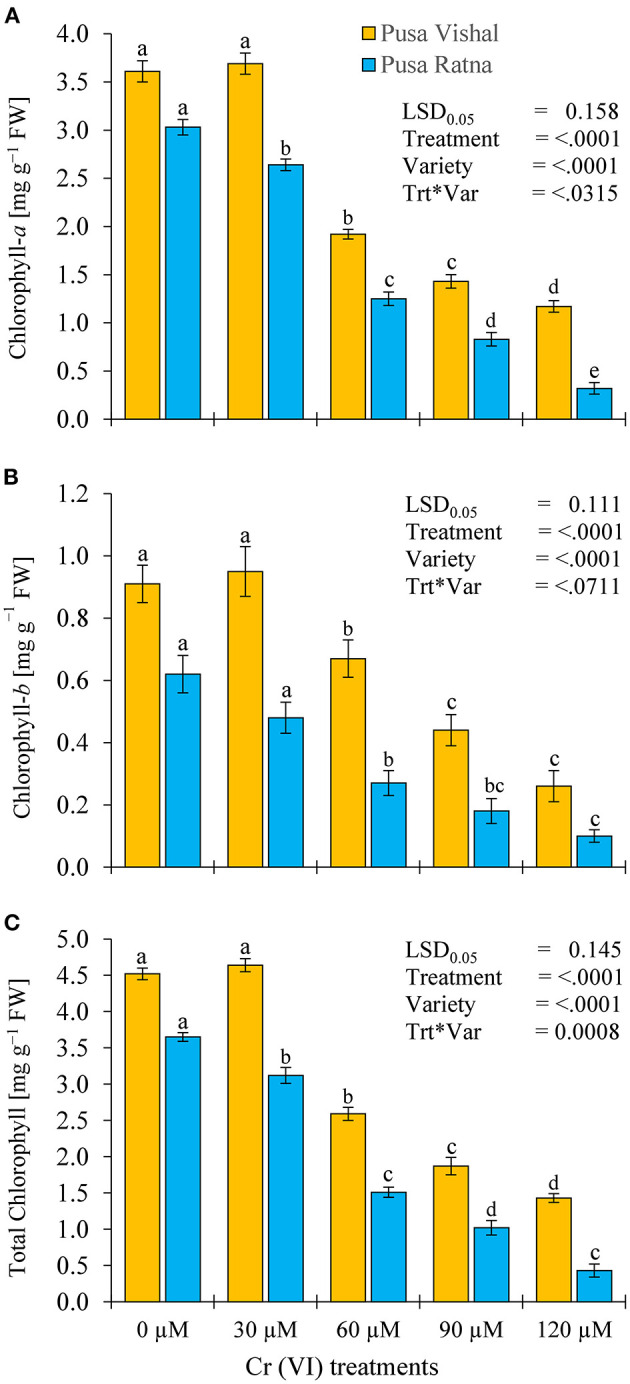
Effect of different (Cr [VI]) treatment levels on chlorophyll contents (mg·g^−1^ FW): **(A)** Chlorophyll-*a*, **(B)** Chlorophyll-*b*, and **(C)** Total Chlorophyll, in two Mungbean cultivars, Pusa Vishal (PV) and Pusa Ratna (PR) 35 days after sowing, in pot conditions. Mean values of three replicates are shown; error bar (± standard error [SE]). Mean values with the same letter don't have significant difference at *p* ≤ 0.05 according to the Least Significant Difference test (LSD_0.05_).

The chlorophyll content of Mungbean plants differed with increasing Cr concentrations (Figure 10). At 120 μM Cr stress condition, reduction in Chl-*a*, Chl-*b* and total Chl contents of PV were 68, 71.43, and 68.24% whereas, PR showed higher reduction of 89.44, 84, and 89%, respectively, when compared to their controls. Measurement of chlorophyll contents (total Chl, Chl-*a*, and Chl-*b*) of both Mungbean cultivars under Cr stress showed a prominent change compared to that of the control, but no significant changes were noted in Chl contents of PV at lower Cr (30 μM) concentration in comparison to the control (Figure 10). Chlorophyll contents were higher in PV than in PR under all Cr treatments, which signifies the higher tolerance to Cr potential in the PV cultivar. Under Cr stress, there were significant variations in N contents in the roots and shoots of both cultivars (Supplementary Figure 3). Under the 120 μM Cr treatment level, the N content of root and shoot in PV decreased by 38% and 42.15%, respectively, compared to the control. Similarly, the N content in root and shoot decreased by 60% and 61% for PR, respectively. However, root and shoot N content of PR were lower than in PV under all Cr treatments.

### Grain Protein Content

**Figure 11:**
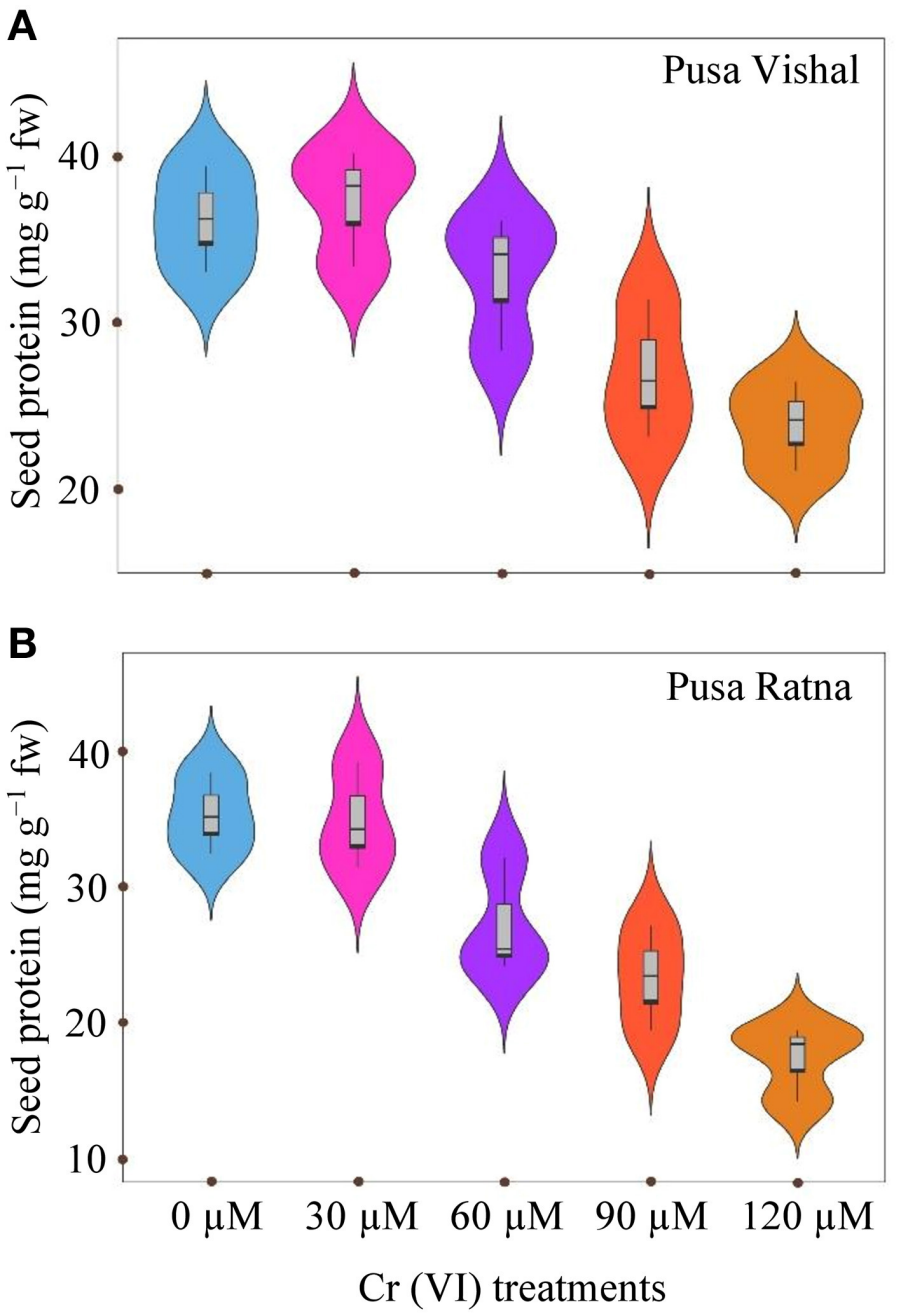
Violin plots screening distribution of values for seed protein content of two mungbean cultivars, **(A)** Pusa Vishal (PV) and **(B)** Pusa Ratna (PR), at 63 days after sowing, when grown in pot conditions under chromium (Cr [VI]) stress.

Increasing the concentration of Cr led to a decrease in seed protein content in both cultivars studied (Figure 11). Seed protein content significantly decreased in both Mungbean cultivars under the 120 μM Cr concentration when compared to the control. Under same treatment condition, reduction in the seed protein was 34.08% in PV and 51% in PR when compared with their control. However, at low concentrations of Cr (30 μM), no significant variation was observed in seed protein contents compared to the control (Figure 11). Seed protein content was greater in PV then in PR under all the Cr treatments.

### Cr Uptake by Different Plant Parts

**Figure 12:**
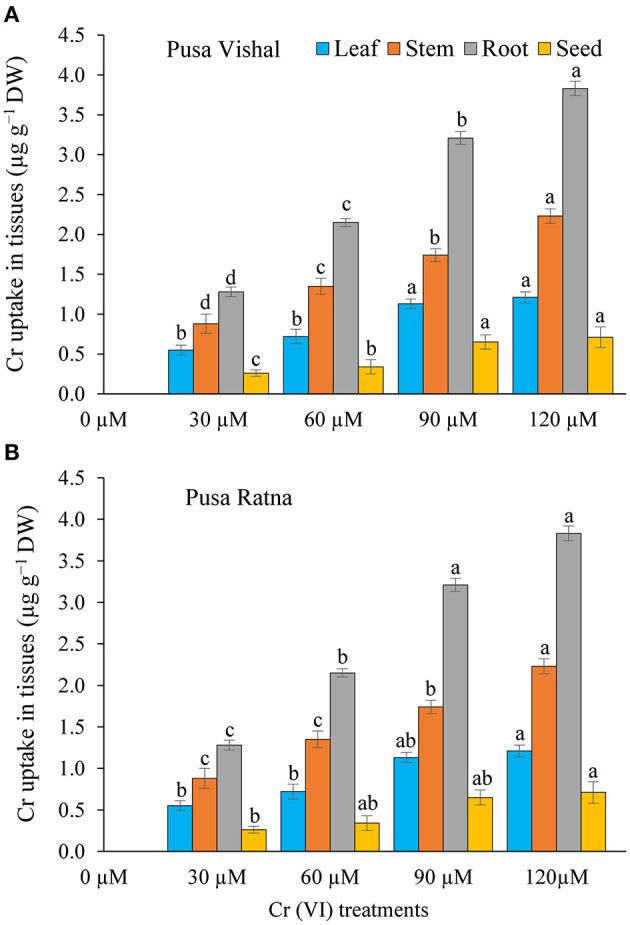
Accumulation of chromium (Cr [VI]) (μg·g^−1^ dry weight [DW]) of organs viz. leaves, stems, roots, and seeds of two Mungbean cultivars, **(A)** Pusa Vishal (PV) and **(B)** Pusa Ratna (PR) in pot conditions under different Cr (VI) treatments. Mean values of three replicates are shown; error bar (± standard error [SE]). Mean values with the same letter don't have significant difference at p ≤ 0.05 according to the Least Significant Difference test (LSD_0.05_).

The total Cr contents in all plant parts of PV and PR cultivars increased with an increase in Cr concentration (Figure 12). Under Cr stress conditions, significant increase of Cr accumultion in root, stems, leaves and seed of PV (3.72, 2.14, 1.18, and 0.64 μg·g^−1^ DW) and PR (3.83, 2.23, 1.21, and 0.71 μg·g^−1^ DW) was observed when compared with control. Cr concentrations in roots and stems of both cultivars increased significantly compared to other parts (leaves and seeds), under all Cr treatments. The varying levels of Cr accumulation under the different Cr concentrations indicated concentration-dependent and cultivar-specific behavior of Mungbean under Cr stress. PV showed the lowest Cr accumulation in the seeds under all Cr treatments, while PR showed the highest Cr accumulation (Figure 12). Therefore, Cr accumulation varied in different parts of plants and was in proportion with Cr concentration, which, in turn, resulted in decreased plant growth traits and crops production. Overall, our results showed that the leaf, stem, root, and seed Cr contents were lower in PV than in PR under Cr (VI) stress.

### Relationship Between Chromium (IV) Uptake and Morphological and Physiological Plant Attributes

**Figure 13:**
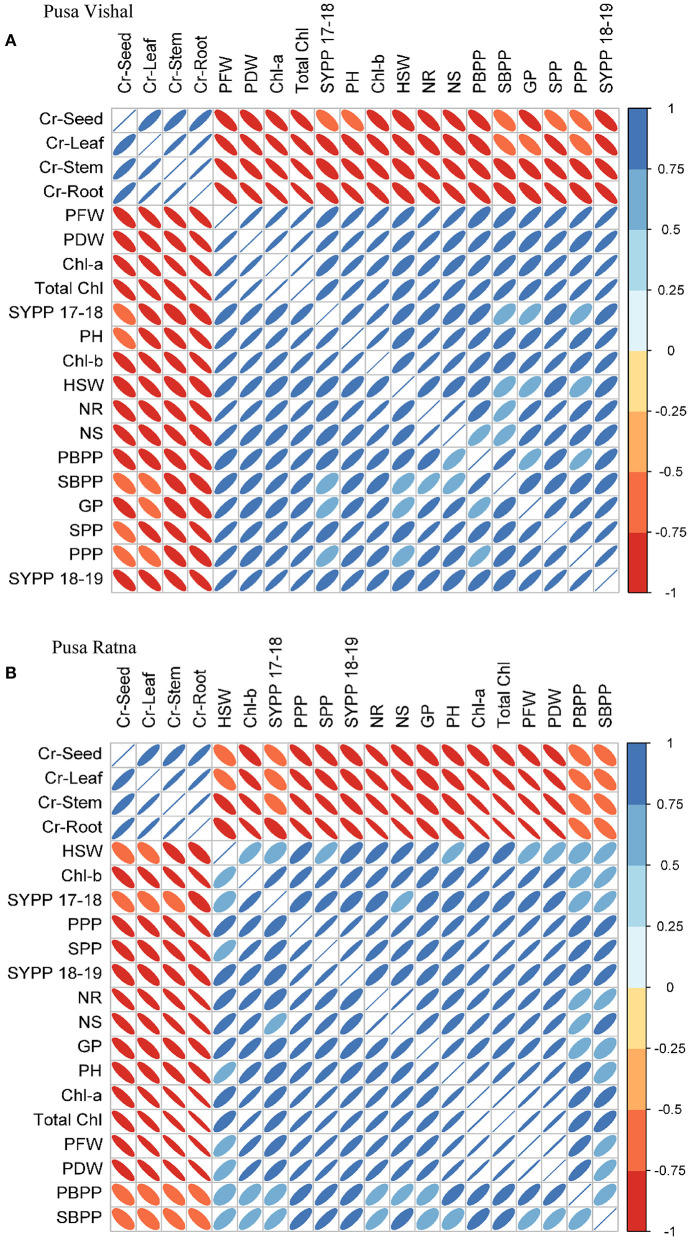
Correlations between different morphological and physiological characteristics and chromium (Cr [IV]) accumulation in different plant parts of **(A)** Pusa Vishal (PV) and **(B)** Pusa Ratna (PR) cultivars in pots. PH (Plant height), PBPP (primary branches per plant), SBPP (secondary branches per plant), PFW (plant fresh weight), PDW (plant dry weight), Chl-a (chlorophyll-a), Chl-b (chlorophyll-b), total chlorophyll, NR (nitrogen root), NS (nitrogen shoot), PPP (pods per plant), SPP (seeds per pod), HSW (hundred seed weight), SYPP 17–18 (seed yield per plant 2017–2018), SYPP 18–19 (seed yield per plant 2018–2019) GP (grain protein) and Cr (Leaf, Stem, Root, Seed).

The relationships between Cr uptake and morpho-physiological characteristics of both Mungbean cultivars are illustrated in Figure 13. PR showed a fairly positive correlation with morphological parameters viz. primary branches per plant, secondary branches per plant, pods per plant, and seed yield per plant, while the physiological characteristics viz. chlorophyll–a, chlorophyll–b, total chlorophyll, seed protein, and N content of the roots and shoots were significantly negatively correlated with Cr under low Cr (VI) treatment. In response to low Cr (VI) concentrations, PV showed significantly positive correlations with morphological parameters (plant height, pods per plant, seeds per pod, plant fresh wight, plant dry weight, and seed yield per plant) and physiological characteristics (chlorophyll–a, chlorophyll–b, total chlorophyll, seed protein, and N content of the roots and shoots) (p < 0.05). However, for both cultivars, there were significant negative correlations in morpho-physiological characteristics under high Cr concentrations (60–120 μM). Therefore, in both Mungbean cultivars, plant growth decreased as Cr concentration increased, with negative correlations. The Cr concentrations in the plant tissues (leaf, stems, roots, and seeds) were negatively correlated with all other plant attributes (Figure 13).

## Discussion

Heavy metal (Cr in particular) pollution in arable land has become a major issue globally and its participation in the ecosystem and environment requires to be resolved. Cr is the most toxic HM, with negative effects on animal and human health, in addition to plant growth, yield, and metabolic activities. In this study, the impact of chromium toxicity on germination, morpho-physiological, biochemical, growth, yield, and yield-related attributes of Mungbean cultivars were investigated, and the parameters were observed to be significantly reduced under Cr stress. Under Cr toxicity, germination and morpho-physiological attributes and biomass production constantly decreased at the seedling stage. Cr adversely affected seed germination of both Mungbean cultivars. Similar studies carried out using high concentrations of Cr have reported decreased seed germination in *Hibiscus esculentus* and in six important legumes (Jun et al., 2009; Amin et al., 2013). The results showed that *Lathyrus odoratus* and *Dumasia villosa* had superior tolerance against Cr stress than the other four crops.

Furthermore, many studies have reported decreases in germination rates under high Cr concentrations in *Vigna radiata* (Rout et al., 1997), *Cucumis melo* (Akinci and Akinci, 2010), *Plantago ovata* (Kundu et al., 2018), and *Solanum lycopersicum* (Brasili et al., 2020; Khan et al., 2020). An increase in Cr level in growth medium led to a decline in the morpho-physiological attributes of Mungbean plants. Phyto-toxic effects of Cr on growth and biomass production have been studied in *Vigna radiate* (Rout et al., 1997; Jabeen et al., 2016), *Brassica oleracea* (Ahmad et al., 2020), *Solanum lycoperscium* (Javed et al., 2021), and *Brassica parachinensis* (Kamran et al., 2021). In this study, high concentration of Cr level (120 μM) resulted in decreases in root, as well as shoot length and biomass of seedlings of both the cultivars (Figure 3), and similar Cr toxicity effects have been observed in *Cicer arietinum* (Singh et al., 2020) and *Vigna radiate* (Jabeen et al., 2016). These results indicate a potential interaction of Cr ions with some essential mineral nutrients in soil, and, in turn, reduces their uptake by plants (del Real et al., 2013). The Cr stress-induced decrease in root length in the present study could be because of Cr accumulation in root cells or due to damage of root tip cells, whereas the decrease in shoot length may be due to ultrastructural damage in leaf mesophyll cells, which ultimately results in decreased shoot development. Similar results have been reported in *Hordeum vulgare* (Ali S. et al., 2013), *Oryza sativa* (Ma et al., 2016; Chen et al., 2017), *Brassica juncea* (Handa et al., 2018), *Arabidopsis thaliana* (Wakeel et al., 2019) and *Cicer arietinum* (Singh et al., 2020). Our results showed that Mungbean could not withstand Cr stress and the Cr-induced reduction in growth traits were greater in PR than in PV.

Oxidative stress induced due to Cr toxicity in both Mungbean cultivars was confirmed based on the increase in EL, MDA and H_2_O_2_ production in the roots and shoots. ROS formation leads to oxidative damage by the disturbance of the equilibrium between the pro-oxidative and the antioxidant defense system. The increased MDA levels in the root and shoot tissues at different concentrations of Cr are considered a sign of oxidative damage due to external Cr toxicity. Furthermore, the increased H_2_O_2_ production and EL resulted in a similar increase in MDA production in plants treated with varying concentrations of Cr, compared to untreated plants, which eroded the equilibrium between ROS production and the antioxidative defense system. Although ROS generation-induced oxidative stress was observed in both the Mungbean cultivars, the effects were higher in PR than in PV, indicating that PV is able to eliminate ROS better and withstand Cr toxicity. Similar results have been reported following research in *Cicer arietinum* (Singh et al., 2020), *Zea mays* (Anjum et al., 2017; Habiba et al., 2019) and *Brassica napus* (Gill et al., 2014, 2015b, 2016b). The tolerant cultivar (PV) had significantly higher proline content than the sensitive cultivar (PR) at the seedling stages and was mainly accumulated in the root and shoot of the plants. Similar findings have been reported in *Cicer arietinum* (Singh et al., 2020). In addition, significantly increased proline contents due to Cr stress have been observed in Zea mays (Adhikari et al., 2020), *Helianthus annuus* (Qadir et al., 2020), and *Sorghum bicolour* (Kumar et al., 2019).

Under Cr stress, antioxidant enzyme activity initially increased at lower Cr concentrations in the leaves of both Mungbean cultivars and then decreased at higher concentrations. Activity of all enzymes in PV leaves increased in the 0–90 μM Cr treatments but decreased in the 120 μM level (Figure 5). Conversely, the activity of all enzymes in PR leaves increased in the 0–60 μM Cr levels but decreased in the 90–120 μM levels (Figure 5). The results displayed that enzyme activity was increased significantly in PV than in PR, suggesting that the former had superior stress tolerance capacity. Enhanced expression of antioxidant genes under Cr (VI) toxicity resulting in marked increase in antioxidant transcripts as a result of activation of plant defense response have been reported in *Zea mays* (Adhikari et al., 2020). The decrease in antioxidant enzyme activity under high Cr levels could be because of severe oxidative stress (Mishra et al., 2006; Zaheer et al., 2020). Generally, under mild HM stress, antioxidant enzyme activity increases, whereas at elevated stress levels, a decrease in enzyme activity is observed (Ali et al., 2018; Mallhi et al., 2019). In this study, POD, SOD, CAT, and APX activity exhibited similar trends. Increase in Cr ions aggravated the oxidant level of the cell that triggered the antioxidant enzymes activities to counter its deleterious effects. However, at higher Cr concentration, all the other pathways such inhibition of enzyme synthesis and assembly also gets affected (Garnier et al., 2006) (Figure 5). This can be explained by damage caused by Cr ions to plant defense machinery. Decrease antioxidant enzyme activity with an increase in Cr toxicity has also been detected in *Brassica napus* (Afshan et al., 2015). Ma et al. (2016) demonstrated that mild Cr in nutrient solutions increased antioxidant activity, while toxicity due to high levels of Cr led to significant reductions in antioxidant enzymes. Therefore, the decrease in antioxidant enzyme activity might be attributed to the high Cr accumulation by Mungbean plants, which might have led to reduced plant defense capacity.

Under Cr stress, plant growth and chlorophyll content were significantly affected in both Mungbean cultivars (Figures 7, 10). In addition to decreased plant growth, chlorophyll content decreased with increasing levels of Cr during the pot experiments. Studies on Cr stress in *Nicotiana tabacum* (Bukhari et al., 2016), *Brassica juncea* (Mahmud et al., 2017; Singh et al., 2017), *Cicer arietinum* (Singh et al., 2020), *Brassica napus* (Gill et al., 2015a), and *Brassica oleracea* (Ahmad et al., 2020) have reported similar results. Our results are also consistent with those in *Vigna radiata* (Jabeen et al., 2016), *Sorghum bicolour* (Kumar et al., 2019), and *Zea mays* (Habiba et al., 2019), in which decreased plant growth and photosynthesis occurred with increasing levels of Cr. Plant growth, biomass production, chlorophyll and N content, and yield characteristics decreased significantly under Cr stress, in the pot experiments. Cr toxicity inhibits cob development in maize cultivars as it induces nutritional imbalances that ultimately disrupt the anabolic pathways, inhibiting normal plant growth (Sharma et al., 2003). N, which is absorbed from soil in the form of nitrate and ammonium, is essential for plant growth and development. The conversion of atmospheric N into ammonia takes place through different chemical processes, among which organic N fixation is the most important (Janczarek et al., 2015; Menéndez et al., 2017). Although N contributes the most to plant growth and development from an early stage, the N content decreased in the root and shoot tissue of both Mungbean cultivars under Cr stress levels, which, in turn, resulted in decreased plant growth and development, as well as decreased pod and seed yield per plant. Singh et al. (2020) reported a significant decrease in root and shoot N contents in *Cicer arietinum*, in addition to grain yield, and seed protein content with increasing levels of Cr. Yield of PV was comparatively superior to that of PR owing to slow Cr uptake and low Cr accumulation. Therefore, considering the low Cr (30 μM) concentration and increased seed productivity in PV, we cannot overlook the potential effect of dilution of Cr content in the seeds. However, overall decrease in pod and seed yield in both Mungbean cultivars could be due to the differential Cr levels in the growth media. The results are consistent with those observed in *Zea mays* (Anjum et al., 2017), *Sorghum bicolour* (Kumar et al., 2019) and *Cicer arietinum* (Singh et al., 2020). Seed yield, pods per plant, HSW, and grain protein content in both Mungbean cultivars decreased with increasing levels of Cr. In pot experiments, carried out in 2017–2018 and 2018–2019, yield per plant increased for both cultivars at 30 μM Cr concentration. Grain protein content was consistent with the observed seed yield under Cr stress. The decrease in grain protein content may attributed to the greater affinity of Cr to protein-ligand, suggesting that potential cellular targets of Cr are enzymes and functional proteins. Present investigation shows Cr ions acted as oxidizing agent and produced free radical that caused oxidative stress in the cell. To mitigate these free radicals, increase in antioxidant enzyme activities were observed in both the cultivars, however it was higher in tolerant cultivars. Further, cell wall of tolerant genotypes showed less damage as compared to sensitive cultivars as elucidated by formation of lower MDA content in tolerant cultivar (Table 1). Both the parameter shows that increase in antioxidant enzyme activities with intact cell wall affirms better Cr tolerance mechanism to plants as also reported by Ali B. et al. (2013) and Gill et al. (2015b).

With an increase in Cr levels, its accumulation also increased in different parts of both the cultivars. Our results are consistent with those observed in *Brassica napus* (Gill et al., 2015b), *Zea mays* (Anjum et al., 2017), *Cicer arietinum* (Singh et al., 2020) and *Brassica oleracea* (Ahmad et al., 2020). In all treatments, higher Cr levels were recorded in the roots than in other tissues (stems, leaves, and seeds) in both the cultivars. Similar results have been reported in *Oryza sativa* (Nagarajan and Ganesh, 2015), *Vigna radiata* (Jabeen et al., 2016), *Brassica campestris* (Zhao et al., 2019), and *Arachis hypogaea* (Zong et al., 2020). Cr accumulation was potentially confined to the roots because of its immobilization in the root cells, thereby affecting plant growth and tolerance under Cr toxicity. Furthermore, Cr content was higher in the roots, stems, leaves, and seeds of PR than those of PV, suggesting that PV has evolved diverse tolerance mechanisms and a greater Cr toxicity tolerance capacity. Similar results have been reported in *Gossypium hirsutum* (Daud et al., 2021), *Cicer arietinum* (Singh et al., 2020) and *Zea mays* (Anjum et al., 2017).

## Conclusions

Chromium concentrations in the environment are increasing owing to industrial and anthropogenic activities. Plants can absorb Cr and experience oxidation cascades, resulting in cell injury. According to the results of the present study, Mungbean can tolerate Cr stress upto 90 μM at seedling stage as it adversely affects plant morpho-physiological and biochemical processes. Therefore, Cr toxicity negatively affected germination and seedling growth attributes of Mungbean, causing increased EL and ROS-induced production of MDA and H_2_O_2_, both in root and shoot tissues. To mitigate the ROS injury, tolerant Mungbean cultivar showed intact cell wall and increase in antioxidant enzyme activity than the sensitive ones. Under the pot experiments, decreases in Mungbean growth, photosynthesis, N and seed protein contents, and yield and yield-related attributes were accompanied by corresponding increases in Cr uptake and accumulation. The morpho-physiological and agronomic characteristics of PV were superior than PR under Cr stress conditions. Further, PR productivity declined significantly under Cr toxicity owing to Cr accumulation at high concentrations in vegetative tissues and increased translocation to the seeds, resulting in reduced yield. Further studies are required to elucidate the molecular basis of Cr interactions in various metabolic pathways. In addition, technological and management approaches to reducing Cr accumulation by plants and, in turn, reduce potential health risks from Cr pollution should be explored.

## Data Availability Statement

The original contributions presented in the study are included in the article/Supplementary Material, further inquiries can be directed to the corresponding authors.

## Author Contributions

DS, NS, CS, and IS conceived the idea and designed research. DS and CS conducted the experiment. DS, CS, and SS did the analysis. DS, CS, SS, and IS analyzed the data and developed the initial full draft of the manuscript. DS, NS, RN, and VY critically reviewed the manuscript draft. All authors have read and agreed to the published version of the manuscript.

## Funding

The University Grants Commission (UGC), New Delhi, India was providing financial support for these research activities.

## Conflict of Interest

The authors declare that the research was conducted in the absence of any commercial or financial relationships that could be construed as a potential conflict of interest.

## Publisher's Note

All claims expressed in this article are solely those of the authors and do not necessarily represent those of their affiliated organizations, or those of the publisher, the editors and the reviewers. Any product that may be evaluated in this article, or claim that may be made by its manufacturer, is not guaranteed or endorsed by the publisher.
