# Supplementary material for: Chromium (VI)-Induced Alterations in Physio-Chemical Parameters, Yield, and Yield Characteristics in Two Cultivars of Mungbean (Vigna radiata L.)
Source: Front Plant Sci. 2021 Sep 29;12:735129. doi: 10.3389/fpls.2021.735129 (PMC8516152; doi:10.3389/fpls.2021.735129)
Supplement: Supplementary file 1 [file Table_1.DOCX]

**Table S1.** Basic organic properties of loam sand used in pot experiment.

| Basic organic properties | loam sand |
| --- | --- |
| Sand (%) | 56.00 |
| Silt (%) | 20.00 |
| Clay (%) | 24.00 |
| Organic matter (%) | 0.37 |
| Phosphorous (P) mg kg ⁻^1^ | 21.40 |
| Potassium (K) mg kg ⁻^1^ | 176.0 |
| Iron (Fe) mg kg ⁻^1^ | 1.24 |
| Nitrogen (N) mg kg ⁻^1^ | 6.54 |
| Electrical conductivity (dsm⁻^1^) | 2.60 |
| Calcium carbonate (CaCO_3_) mg kg ⁻^1^ | 1.54 |
| Copper (Cu) mg kg ⁻^1^ | 0.002 |
| Manganese (Mn) mg kg ⁻^1^ | 0.045 |
| Zinc (Zn) mg kg ⁻^1^ | 0.07 |
| Chromium (Cr) | BDL |
| Lead (Pb) | BDL |
| Cadmium (Cd) mg kg ⁻^1^ | 0.001 |
| pH | 7.31 |
| Aluminum (Al) mg kg ⁻^1^ | 0.004 |

Note: - BDL- Below Detection Limit
