# Supplementary figures and images for: Chromium (VI)-Induced Alterations in Physio-Chemical Parameters, Yield, and Yield Characteristics in Two Cultivars of Mungbean (Vigna radiata L.)
Source: Front Plant Sci. 2021 Sep 29;12:735129. doi: 10.3389/fpls.2021.735129 (PMC8516152; doi:10.3389/fpls.2021.735129)

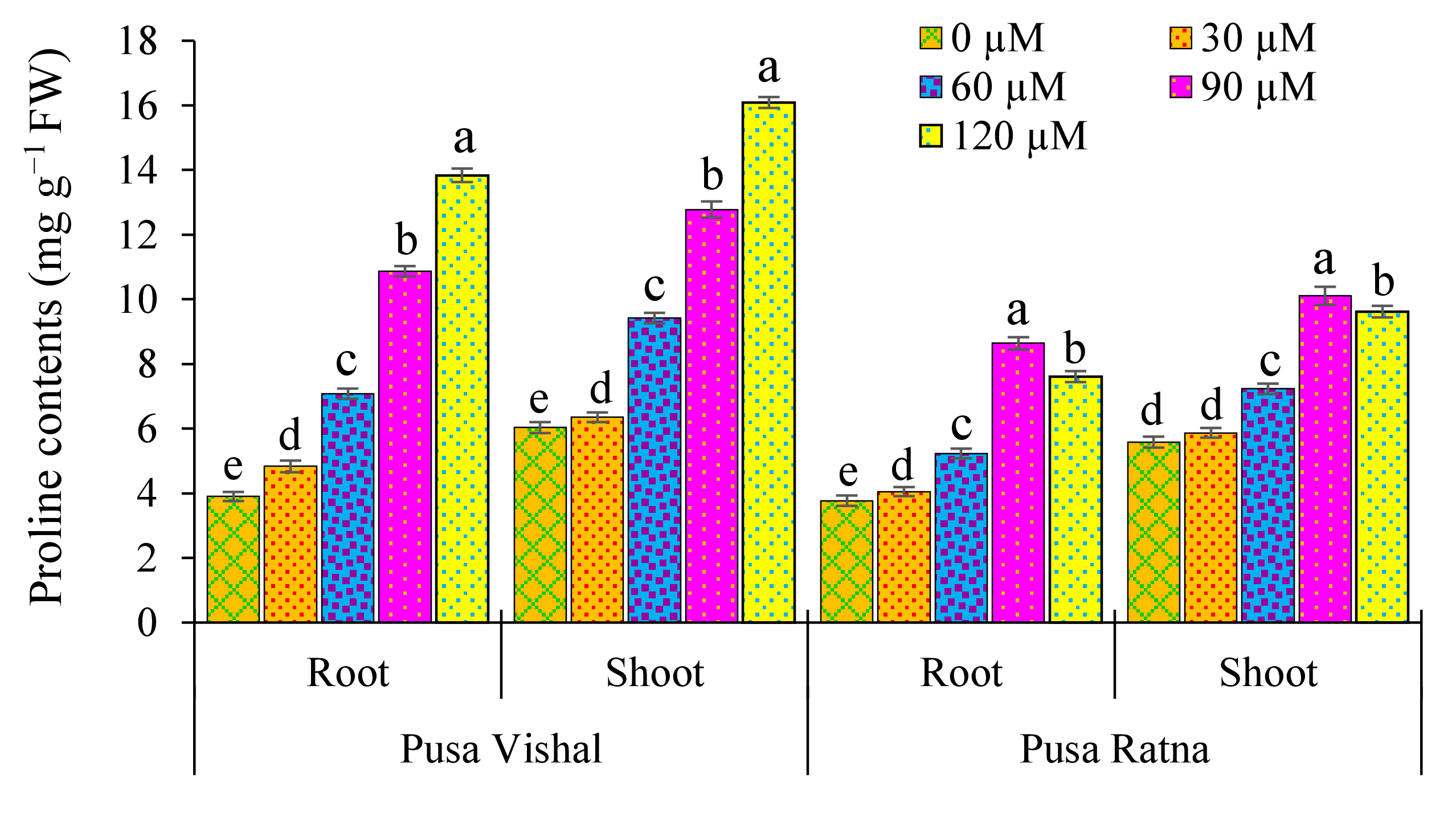

Supplement: Supplementary file 2 [file Image_1.TIF]

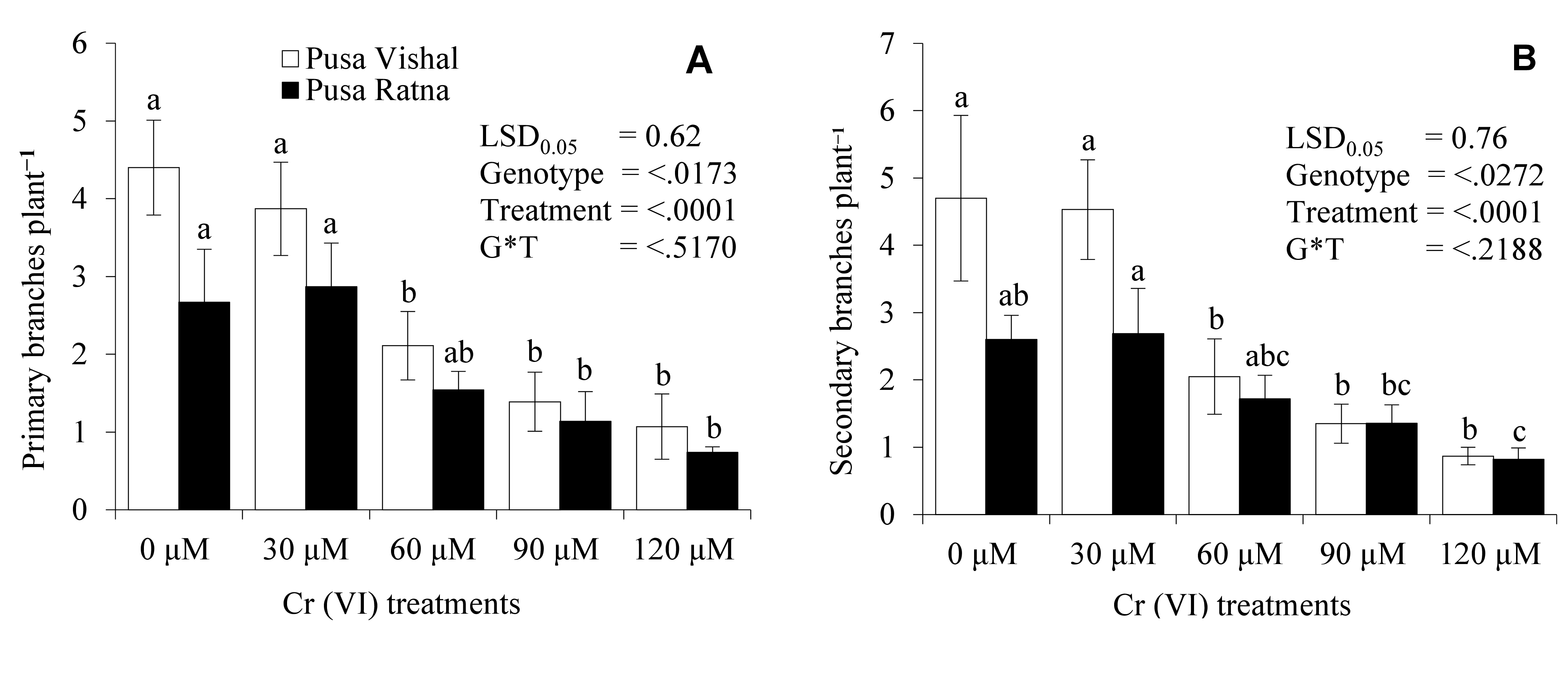

Supplement: Supplementary file 3 [file Image_2.TIF]

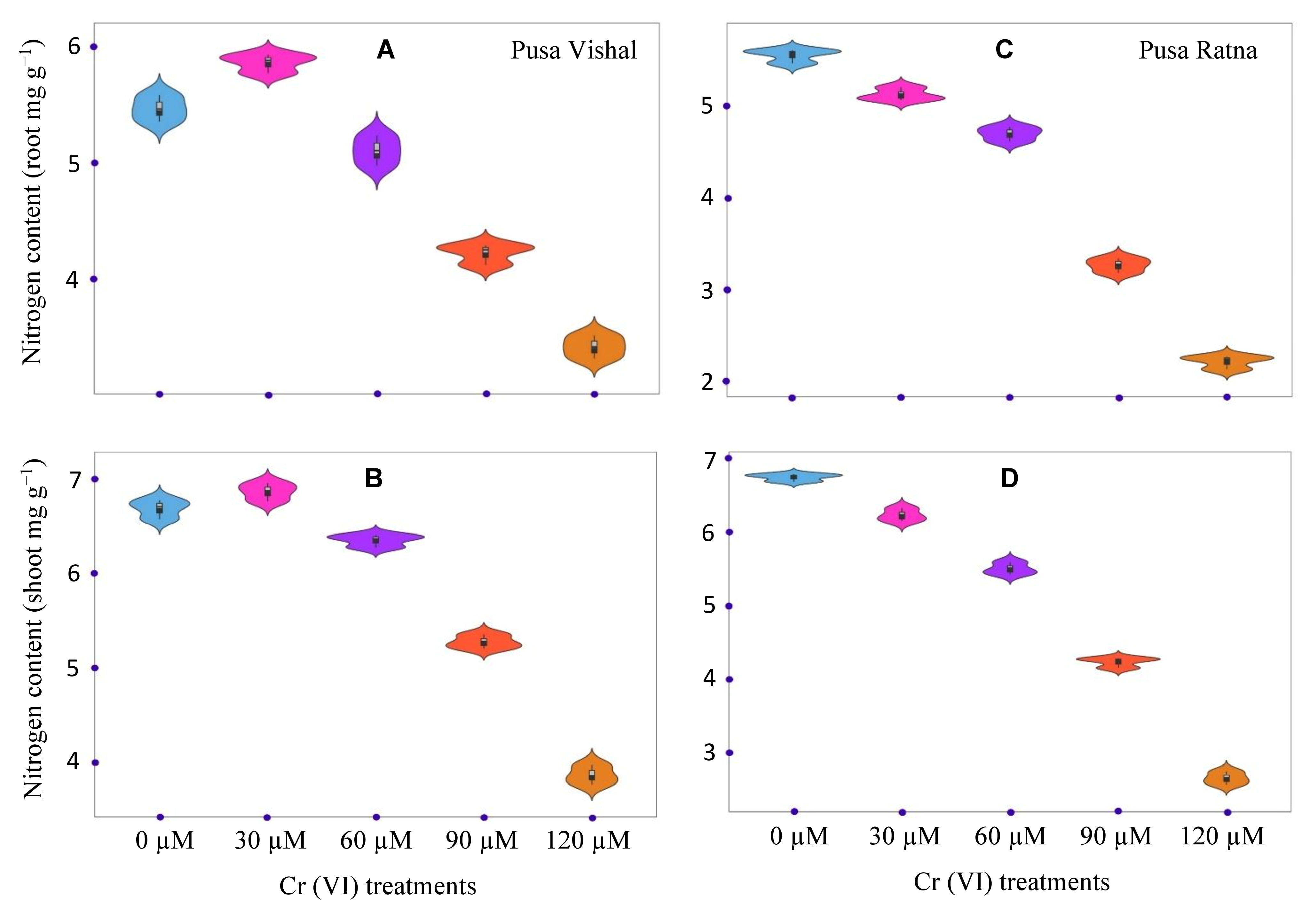

Supplement: Supplementary file 4 [file Image_3.TIF]
